# Effect of deep brain stimulation on motor complications in Parkinson’s disease: a systematic review and meta-analysis

**DOI:** 10.3389/fnhum.2025.1684229

**Published:** 2025-12-18

**Authors:** Hu Xu, Xing Wan, Qi Tu, Huahui Chen, Minfeng Tong, Zhijian Xu, Dandan Cai

**Affiliations:** Jinhua Hospital Affiliated to Zhejiang University (Jinhua Municipal Central Hospital), Zhejiang, China

**Keywords:** deep brain stimulation, Parkinson’s disease, motor complications, UPDRS-III, meta-analysis

## Abstract

**Background:**

Deep brain stimulation (DBS) significantly improves tremor, rigidity, bradykinesia, and dyskinesia for patients with Parkinson’s disease (PD), but gait and speech remain inconsistent. These discrepancies underscore the need for a systematic, quantitative synthesis of existing data to clarify the impact of DBS across different motor domains.

**Objective:**

To systematically evaluate the effects of DBS on motor symptoms in PD by analyzing UPDRS-III scores and conducting subgroup analyses based on stimulation target, stimulation type, and medication status.

**Methods:**

A literature search was conducted to identify relevant studies on clinical trials and observational studies reporting pre- and post-DBS motor assessments in PubMed, Embase, the Cochrane Central Register of Controlled Trials (CENTRAL), Ovid MEDLINE, and Web of Science from inception to 15 December 2024.

**Results:**

A total of 35 studies comprising 1,082 PD patients were included. The pooled analysis demonstrated a significant improvement in overall UPDRS-III scores post-DBS (WMD: = −1.09, 95% CI: −1.32 to −0.87, *p* < 0.05). Subgroup analyses showed consistent improvements across tremor, rigidity, akinesia, bradykinesia, dyskinesia, and axial symptoms, regardless of stimulation target or medication state. UPDRS Part IV scores also significantly improved, reflecting reduced motor complications. However, speech function remained unchanged, and UPDRS Part I scores initially showed no significant improvement, though significance emerged after removing sources of heterogeneity.

**Conclusion:**

DBS significantly improves overall motor function, particularly tremor, rigidity, and bradykinesia. However, its effects on gait and speech remain inconsistent, which shows the need for further research to refine patient selection and optimize stimulation parameters. These findings provide valuable insights into the therapeutic impact of DBS in PD management.

## 1 Introduction

Parkinson’s disease (PD), a progressive neurological disorder with motor and non-motor symptoms, profoundly affects patients’ quality of life (Hague et al., 2005; Sprenger and Poewe, 2013). PD has become a growing public health concern, China and East Asia report some of the highest age-standardized incidence and prevalence rates worldwide, with a marked increase in disease burden. Between 1990 and 2021, PD incidence, prevalence, and mortality in Asia surged by nearly 200, 280, and 110%, respectively (Chang et al., 2024; Xu et al., 2024). Bradykinesia, stiffness, tremor, and postural instability are the major motor symptoms, which are largely brought on by the loss of dopaminergic neurons in the substantia nigra (Palakurthi and Burugupally, 2019). Even though levodopa is still the most effective medication, prolonged use can cause motor issues like fluctuations and dyskinesia (Katzenschlager and Lees, 2002). In response, surgical interventions like deep brain stimulation (DBS) have been developed to modulate neural activity in key motor circuits and alleviate symptoms (Lin et al., 2022; Hariz and Blomstedt, 2022; Moshayedi et al., 2024). Despite its widespread clinical use, there remain gaps in understanding the extent of its benefits across different motor dysfunctions.

DBS modifies malfunctioning neural circuits by mainly focusing on the globus pallidus internus (GPi) or the subthalamic nucleus (STN) (Au et al., 2021; Zhang et al., 2021). The STN-DBS approach is thought to work by inhibiting excessive excitatory output from the STN, thereby reducing pathological hyperactivity in the basal ganglia-thalamocortical loop, which is responsible for many motor impairments in PD (Galvan et al., 2015). This modulation leads to improvements in bradykinesia, rigidity, and tremor, while also enabling significant reductions in dopaminergic medication, which can help mitigate levodopa-induced dyskinesias (Wichmann and DeLong, 2011). On the other hand, GPi-DBS is primarily associated with a direct suppression of excessive inhibitory output from the GPi, thereby restoring balanced motor control and reducing both primary motor symptoms and levodopa-induced dyskinesia (Nambu and Chiken, 2014; Chiken and Nambu, 2013). While both targets have demonstrated substantial benefits, their relative advantages and disadvantages remain a subject of ongoing clinical investigation. To objectively measure the influence of DBS on PD motor symptoms, the Unified Parkinson’s Disease Rating Scale (UPDRS) is extensively utilized (Movement Disorder Society Task Force on Rating Scales for Parkinson’s Disease, 2003), with its third part (UPDRS-III) being the most critical for evaluating motor function. UPDRS-III is a clinician-rated scale that quantifies the severity of motor impairments across multiple domains, including tremor, rigidity, bradykinesia, postural stability, gait disturbances, and speech dysfunction. It provides a standardized framework for measuring disease progression and treatment efficacy. Improvements in UPDRS-III scores following DBS indicate a reduction in motor disability and an enhancement of overall functional capacity (Kahn et al., 2019).

Additionally, while DBS has been shown to effectively reduce tremor, rigidity, and bradykinesia, its effects on more complex motor symptoms such as gait dysfunction, speech impairment, and axial symptoms remain controversial. Some studies suggest that DBS may exacerbate speech problems or lead to postural instability, whereas others report improvements or no significant changes (Deuschl et al., 2006; Rossi et al., 2018). A meta-regression analysis in 2010 including 12 studies (nine with STN-DBS and three with GPi-DBS) on postural instability and gait disorder (PIGD) in Parkinson’s disease demonstrated that PIGD tends to return to the preoperative level within two years after STN-DBS under the medication-on condition (St George et al., 2010). While the chronic decline in efficacy may reflect disease progression and the associated deterioration of postural control, early gait worsening following DBS is often attributed to suboptimal stimulation parameters. Importantly, gait and speech impairments in Parkinson’s disease are not solely caused by dopaminergic nigrostriatal degeneration—which can be effectively modulated by DBS—but also by widespread network dysfunctions involving nondopaminergic systems such as the pedunculopontine nucleus, cerebellothalamic pathways, and cortical-basal ganglia loops. This network-level complexity likely underlies the limited responsiveness of gait and speech functions to conventional DBS approaches. These discrepancies underscore the need for a systematic, quantitative synthesis of existing data to clarify the impact of DBS across different motor domains.

This systematic review and meta-analysis aim to provide an updated and comprehensive evaluation of the effects of deep brain stimulation (DBS) on motor and related complications in Parkinson’s disease. Unlike earlier meta-analyses conducted nearly two decades ago (Kleiner-Fisman et al., 2006), this study incorporates a substantial body of new evidence published in recent years. By analyzing the Unified Parkinson’s Disease Rating Scale (UPDRS) across all parts (I–IV) and detailed subdomains—including tremor, rigidity, akinesia, dyskinesia, gait, axial symptoms, and speech function—this study offers a multidimensional assessment of DBS efficacy. Subgroup analyses based on medication status (med-on vs. med-off), stimulation type (bilateral vs. unilateral), and target site (GPi vs. STN) further clarify the determinants of treatment variability. The findings aim to refine clinical decision-making and support the development of personalized DBS strategies in Parkinson’s disease management.

## 2 Methods

### 2.1 Search strategy and selection criteria

The systematic review followed the Cochrane Handbook for Systematic Reviews of Interventions and adhered to PRISMA reporting guidelines (Parums, 2021). A literature review was conducted across multiple databases, including PubMed, Embase, the Cochrane Central Register of Controlled Trials (CENTRAL), Ovid MEDLINE, and Web of Science. The search was confined to studies published in English and available until December 15, 2024. The searches were performed using the medical subject headings (MeSH) phrases, “Deep Brain Stimulation” and “Parkinson” and “Dyskinesia.” The search approaches used for all databases are showed in Supplementary Table 1.

This meta-analysis established selection criteria to ensure a systematic and rigorous assessment of DBS effects on UPDRS-III and its subcomponents. Titles and abstracts were initially screened to remove duplicates and exclude studies that did not investigate DBS outcomes in Parkinson’s disease. Full-text publications were then screened for eligibility using preset inclusion criteria. Studies were included, if they (1) reported quantitative data on motor symptoms or complications (e.g., UPDRS-III scores, dyskinesia, tremor, rigidity, bradykinesia) before and after DBS treatment, (2) provided sufficient statistical data (mean, standard deviation, or effect size) for meta-analysis, and (3) employed a case–control, cohort, or randomized controlled trial (RCT) design. Studies were excluded if they (1) lacked empirical data, (2) did not specify stimulation targets or medication status, (3) were review articles or case reports, or (4) were not published in English. The final selection included studies that met all eligibility criteria and were suitable for data extraction and quality assessment using standardized tools.

### 2.2 Data extraction

EndNote 20 was used for reference management and study organization. After removing duplicates, two independent researchers systematically screened the titles and abstracts of the remaining studies. Only studies meeting the inclusion criteria, with mutual agreement between both researchers, were included in the meta-analysis. In cases of disagreement, discussions were held, and if a consensus could not be reached, a third reviewer provided the final decision.

A standardized data extraction template was employed to ensure consistency and accuracy across studies. Key study characteristics, including authorship, year of publication, and number of participants, were recorded. Extracted participant data encompassed demographics such as age, gender, disease duration, and baseline health status. For the evaluation of DBS outcomes, quantitative data on motor complications were collected, encompassing UPDRS-III scores, tremor, rigidity, bradykinesia, dyskinesia, axial symptoms, and other relevant movement impairments. Comparator groups, stimulation targets, medication status, and follow-up durations were also documented. Quantitative data were extracted as mean ± standard deviation (SD) from text, tables, or additional materials.

### 2.3 Assessment of risk of bias

The Revman 5.4.1 risk of bias criteria, which comprised seven items—randomization sequence generation (selection bias), allocation concealment (selection bias), blinding of personnel and participants (performance bias), blinding of outcome assessment (detection bias), incomplete outcome data (attrition bias), selective reporting (reporting bias), and other bias—were used to assess the methodological quality of the included studies (Mao et al., 2024). To produce an overall bias judgment for the specific study outcome under consideration, each item was graded as “low risk,” “unclear risk,” or “high risk” based on the signaling questions. The methodological quality was assessed independently by two reviewers. A third author was recruited to resolve discrepancies in the reviewers’ evaluations.

### 2.4 Statistical analysis

A random-effects REML model was applied to address study variability in the pooled analysis of mean differences for each motor complication outcome pre- and post-DBS treatment. Heterogeneity was evaluated using the *I*^2^ statistic, with values above 50% indicating substantial inconsistency. A funnel plot was used to assess publication bias, and Egger’s regression and Begg’s rank correlation tests were used to further quantify it; a *p*-value of less than 0.05 was deemed statistically significant. To explore potential sources of heterogeneity, a Galbraith plot was generated. A leave-one-out sensitivity analysis was performed when the Galbraith plot indicated that fewer than 25% of studies contributed to heterogeneity, reassessing the overall effect after their exclusion. Subgroup analyses were conducted to evaluate the impact of key factors, including medication status (med on vs. med off), stimulation type (bilateral vs. unilateral), and DBS target site (GPi vs. STN), to determine their influence on treatment outcomes. All statistical analyses were conducted using Stata software, version 18.0.

## 3 Results

### 3.1 Study inclusion

**Figure 1:**
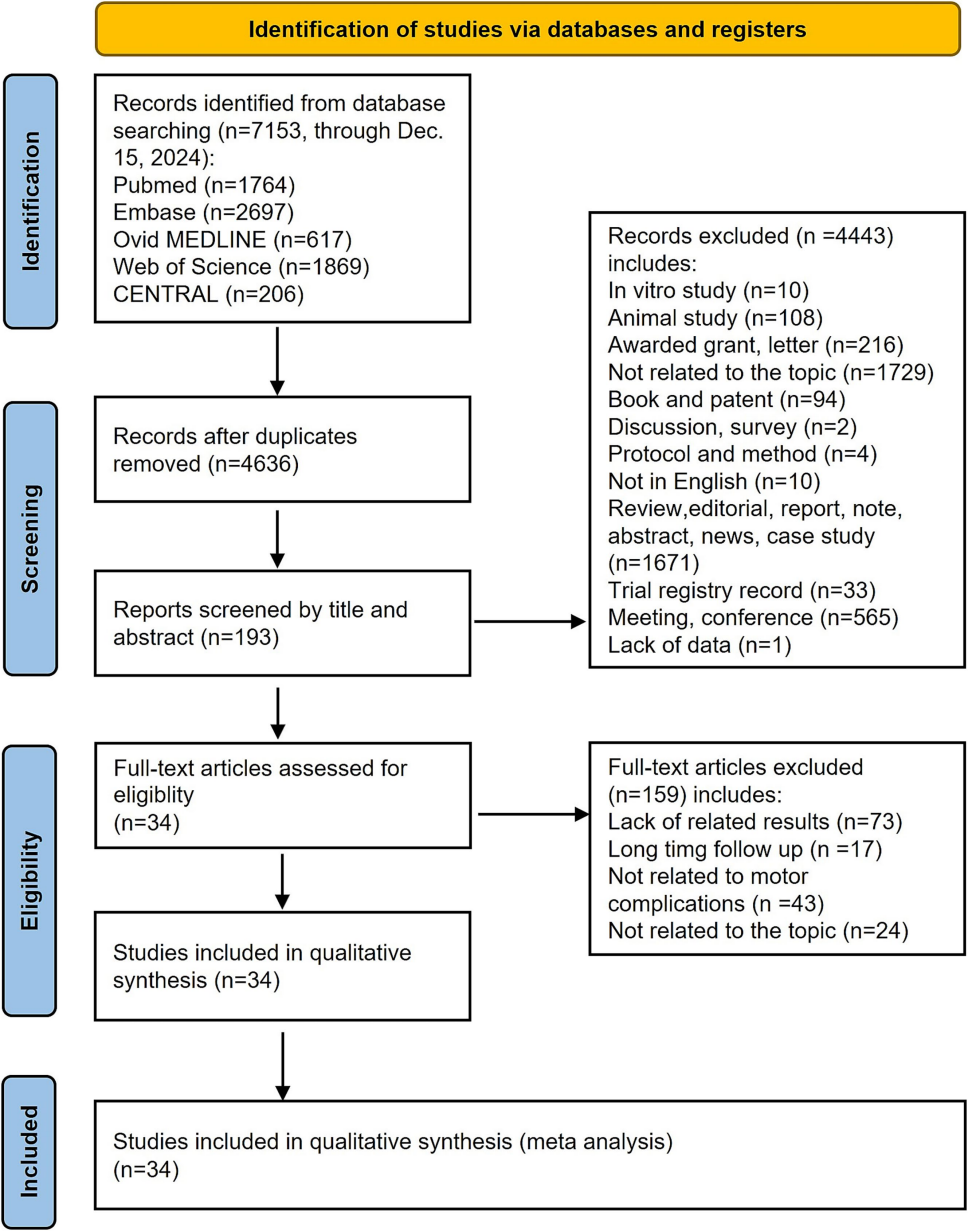
Flowchart of study identification, screening, eligibility, and inclusion according to PRISMA guidelines.

A comprehensive search identified 7,153 records from databases and relevant reference lists. After duplicate removal, 4,636 unique publications remained and were screened by title and abstract. Of these, 4,443 were excluded for not meeting the predefined inclusion and exclusion criteria. Ultimately, 193 full-text articles were assessed for eligibility, with 35 meeting all inclusion criteria for the final meta-analysis, as shown in Figure 1.

### 3.2 Characteristics and quality assessment of studies

This meta-analysis included 35 studies comprising 1,082 Parkinson’s disease patients who underwent DBS. Participants ranged in age from 20 to 80 years, with reported disease duration and Hoehn & Yahr stages indicating varied severity. Most studies compared DBS targeting the STN and GPi using unilateral or bilateral stimulation, and documented gender ratios. Unified Parkinson’s Disease Rating Scale (UPDRS) scores were consistently reported pre- and post-DBS, including UPDRS-III under medication-on and medication-off conditions, enabling subgroup analyses, while several studies also reported Parts I, II, and IV (Table 1).

**Table 1:** Clinical features of PD patients reported in included DBS studies.

Reference	Publish year	DBS	N	Age (year)	Age at surgery (year)	Mean PD duration (year)	Modified Hoehn & Yahr stage	Gender (male:female)	UPDRS total (med-off)	UPDRS total (med-on)
Kelly et al. (2010)	2010	STN-DBS (unilat and bilat)	8	51.9 ± 8.7	NA	10.1 ± 3.5	4.0 ± 0.9/2.5 ± 0.5	3:1	49.2 ± 17.2	23.8 ± 11.8
Chiou (2016)	2016	STN-DBS	72	40–78	61.1 ± 9.7	7.9 ± 3.7	3	2:1	67.3 ± 23.5	48.7 ± 20.3
Anderson et al. (2005)	2005	GPi or STN DBS	20	20–80	NA	10.3 ± 2 /15.6 ± 5 (Gpi/STN)	3–4	NA	92 ± 31/88 ± 20 (Gpi/STN)	44 ± 27/43 ± 15 (Gpi/STN)
Gurutz et al. (2003)	2003	STN-DBS (unilat)	8	65 ± 6	NA	13.7 ± 3.5	NA	1:1	NA	NA
Merello et al. (1999)	1999	GPi (*n* = 7) or STN (*n* = 6) DBS (unilat)	13	59 ± 6 /55 ± 10 (Gpi/STN)	NA	12.5 ± 5 /13.5 ± 4.5 (Gpi/STN)	1–5	9:4	NA	27.5 ± 9.4/29.5 ± 10.5 (Gpi/STN)
Han-Joon et al. (2009)	2009	STN-DBS (unilat)	8	57 ± 10	NA	6.8 ± 2.8	NA	5:3	NA	NA
Bonenfant et al. (2017)	2007	Gpi-DBS	25	60 ± 8	NA	12.5 ± 6.25	3.2 ± 1.1	13:12	NA	NA
Burchiel et al. (1999)	1999	GPi (*n* = 4) or STN (*n* = 6) DBS (bilat)	10	47 ± 11 /63 ± 12 (Gpi/STN)	NA	10.6 ± 2/13.6 ± 5 (Gpi/STN)	2–4	7:3	113 ± 42/85 ± 2 (Gpi/STN)	66 ± 35/49 ± 16 (Gpi/STN)
Castrioto et al. (2011)	2011	STN-DBS (bilat)	18	40 ± 7	52.9 ± 7.9	13.4 ± 4.8	NA	2:1	NA	NA
Thobois et al. (2002)	2002	STN-DBS (bilat)	18	57 ± 6	NA	13.5 ± 4.4	3.6 ± 0.7	1:1	NA	NA
Rodriguez-Oroz et al. (2005)	2005	GPi (*n* = 49) or STN (*n* = 20) DBS (bilat)	69	NA	59.8 ± 9.8/55.8 ± 9.4 (Gpi/STN)	3.8 ± 0.6/3.9 ± 0.7 (Gpi/STN)	4.3 ± 0.8/4.0 ± 0.8 (Gpi/STN)	25:24/13:7 (Gpi/STN)	56.7 ± 15.7/51.7 ± 13.6 (Gpi/STN)	22.8 ± 10.4/18.6 ± 10.3 (Gpi/STN)
Walker et al. (2009)	2009	STN-DBS (unilat)	37	60 ± 11	NA	NA	NA	17:8	34.1 ± 1.6	NA
Shen et al. (2023)	2023	GPi DBS (bilat)	55	51 ± 8	64.6 ± 8	13.3 ± 4.8	2.6 ± 0.5	32:23	52.4 ± 13.1	24.4 ± 8.5
Shang-Ming et al. (2015)	2015	STN-DBS (bilat)	62	61 ± 2	NA	8.2 ± 0.8	2–5	43:19	40.5 ± 4.0	27.1 ± 3.6
Doshi et al. (2003)	2003	STN-DBS (bilat)	23	58 ± 9	NA	/	3–4	18:5	NA	NA
Du et al. (2022)	2022	STN-DBS (bilat)	51	52 ± 9	62.47 ± 7.73	10.24 ± 4.31	3	30:21	47.55 ± 15.61	24.27 ± 13.58
Erola et al. (2006)	2006	STN-DBS (bilat)	29	60 ± 8	NA	13 ± 7	2.9 ± 0.7	20:9	NA	NA
Krause et al. (2022)	2022	STN-DBS (bilat)	46	NA	51.0 ± 8.8	12.9 ± 5.7	NA	27:19	NA	NA
Katayama et al. (2001)	2001	STN-DBS (bilat)	7	61 ± 10	NA	7.6 ± 5.2	3–4	4:3	NA	NA
Krack et al. (1998)	1998	GPi (*n* = 5) or STN (*n* = 8) DBS (bilat)	13	<40	51 ± 4/51 ± 10 (Gpi/STN)	16 ± 4/16 ± 5 (Gpi/STN)	4–5	4:1/5:3 (Gpi/STN)	53.6 ± 10.4/57.5 ± 14.5 (Gpi/STN)	23.2 ± 4/18.2 ± 0.6 (Gpi/STN)
Krystkowiak et al. (2003)	2003	STN-DBS (bilat)	10	57 ± 8	NA	13 ± 5	3–5	7:3	NA	NA
Krystkowiak et al. (2001)	2001	GPi-DBS (bilat)	5	57 ± 9	/	15.8 ± 3.8	4	2:3	NA	NA
Liang et al. (2006)	2006	STN-DBS (bilat)	27	47 ± 9	59.5 ± 9	11.9 ± 3.6	3.5 ± 0.9	2:1	67 ± 18.2	29.2 ± 13.1
Loher et al. (2002)	2002	GPi-DBS (unilat and bilat)	19	49 ± 7/45 ± 8 (unilat /bilat)	65.1 ± 5.4/64.6 ± 9.9 (unilat /bilat)	16.7 ± 6.3 (unilat) 19.6 ± 7.9 (unilat /bilat)	4.2 ± 0.8/4.4 ± 0.7 (unilat /bilat)	2:2/1:1 (unilat /bilat)	57.2 ± 13.7/63.4 ± 17.4 (unilat /bilat)	29.1 ± 6.5/37.6 ± 17.3 (unilat /bilat)
Merello et al. (2001)	2001	GPi-DBS (bilat)	6	55 ± 9	NA	10 ± 4.9	4	5:1	28.5 ± 11.5	NA
Aristide et al. (2015)	2015	STN-DBS	198	44 ± 8	55.7 ± 7.1	11.9 ± 2.8	<3	61.8:38.2	NA	NA
Pahwa et al. (2003)	2003	STN-DBS (bilat)	33	59	NA	11.8	3.1 ± 0.5	7:4	NA	NA
Stergaard et al. (2002)	2002	STN-DBS (bilat)	26	59 ± 8	NA	15 ± 5	3.7 ± 0.8	21:5	51.3 ± 12.1	23.5 ± 15.8
Pinter et al. (1999)	1999	STN-DBS (bilat)	9	57 ± 10	NA	11.3 ± 4.3	4.1 ± 0.4	2:1	NA	NA
Volkmann et al. (2001)	2001	GPi (*n* = 11) or STN (*n* = 16) DBS (bilat)	27	57 ± 9/60 ± 10 (Gpi/STN)	NA	10.5 ± 2.7/13.1 ± 5.9 (Gpi/STN)	NA	NA	NA	NA
Rabie et al. (2016)	2016	STN-DBS (bilat)	20	61 ± 9	NA	11.7 ± 6.4	3.6 ± 0.7	7:3	40.0 ± 12.4	NA
Wider et al. (2008)	2008	STN-DBS (bilat)	37	65 ± 8	NA	14.4 ± 4.9	NA	3:2	NA	NA
Sobstyl et al. (2014)	2014	STN-DBS (bilat)	16	54 ± 7	63.5 ± 5.2	NA	3.4 ± 0.6	11:5	40.9 ± 10.7	24.0 ± 3.5
Tandra et al. (2020)	2020	STN-DBS (bilat)	40	48 ± 10	55.5 ± 9.7	7.3 ± 2.8	NA	NA	NA	NA
Tsai et al. (2013)	2013	STN-DBS (bilat)	17	32 ± 7	44.8 ± 10.6	12.5 ± 7.5	NA	15:2	NA	NA

NA indicates data not reported in the original study.

**Figure 2:**
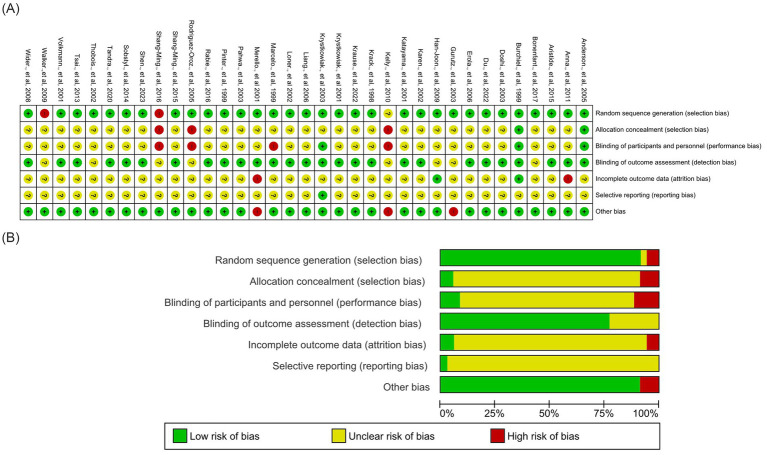
Risk of bias in the included studies. **(A)** Risk of bias summary: authors’ individual assessments of each risk-of-bias domain for every included study. **(B)** Distribution of the authors’ judgments for each bias domain presented as percentages across all included studies. Domains include sequence generation, allocation concealment, blinding of participants and outcome assessors, incomplete outcome data, selective reporting, and other potential sources of bias.

Methodological quality assessment using the RevMan 5.4.1 risk-of-bias tool revealed generally low risk across most domains, but allocation concealment and participant blinding were often unclear or high risk due to the nature of surgical interventions. Detection bias was lower because outcome assessments were frequently blinded. Attrition and reporting biases were occasionally unclear, reflecting missing data or incomplete reporting, and some studies showed other biases related to small sample sizes or design limitations. These methodological factors should be considered when interpreting the pooled results (Figure 2).

### 3.3 Meta-analysis of deep brain stimulation’s effects on UPDRS-III score

**Figure 3:**
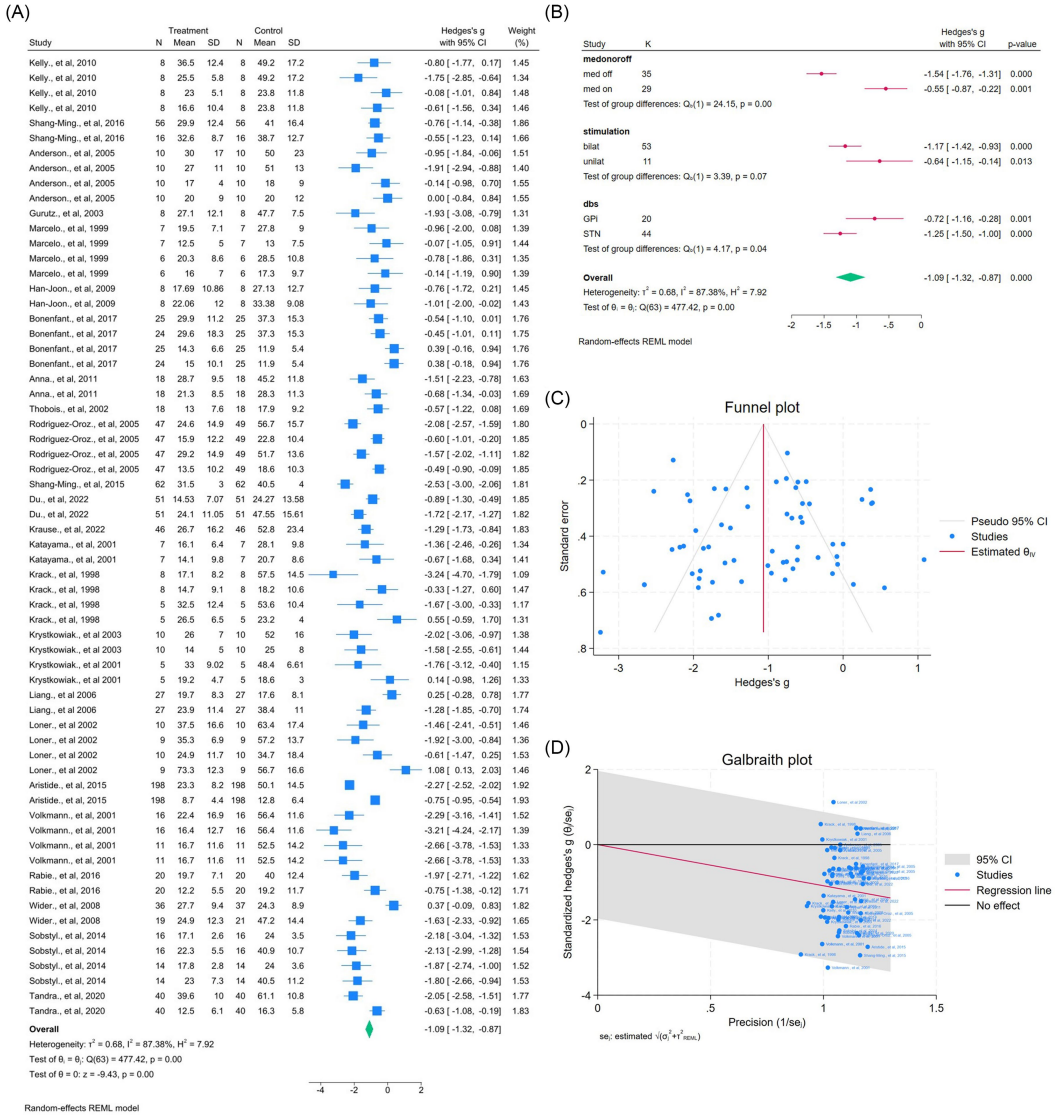
Meta-analysis of the effects of Deep Brain Stimulation (DBS) on UPDRS-III motor scores in Parkinson’s disease (PD). **(A)** Forest plot illustrating the overall effect of DBS on UPDRS-III total scores before and after surgery. **(B)** Subgroup analyses stratified by medication status (med-on vs. med-off), stimulation type (bilateral vs. unilateral), and stimulation target (GPi vs. STN). **(C)** Funnel plot assessing potential publication bias using pseudo 95% confidence limits. **(D)** Galbraith plot identifying potential sources of between-study heterogeneity. The pooled weighted mean difference (WMD) and 95% confidence intervals (CIs) are depicted by diamond markers. The random-effects model (REML estimation) was applied to account for variability across studies. *I*^2^ quantifies the percentage of total variation due to heterogeneity rather than chance, while *p* < 0.05 was considered statistically significant. Study-specific effect sizes (*θᵢ*) were tested for homogeneity (*H*₀: *θᵢ* = *θⱼ*) using the chi-squared test. DBS, deep brain stimulation; GPi, globus pallidus internus; STN, subthalamic nucleus; UPDRS, unified Parkinson’s disease rating scale.

Across 25 studies (64 outcomes), DBS significantly improved overall UPDRS-III scores (WMD = −1.09, 95% CI –1.32 to −0.87; *p* < 0.01; Figure 3A), despite substantial heterogeneity (*I*^2^ = 87.4%, *p* < 0.01). Subgroup analyses revealed differences by medication status, stimulation laterality, and target (all *p* < 0.0001; Figure 3B). No publication bias was detected (Figure 3C). Two outliers contributed to heterogeneity, but exclusion had minimal effect (Figure 3D, Supplementary Figure S1).

#### 3.3.1 Tremor

**Figure 4:**
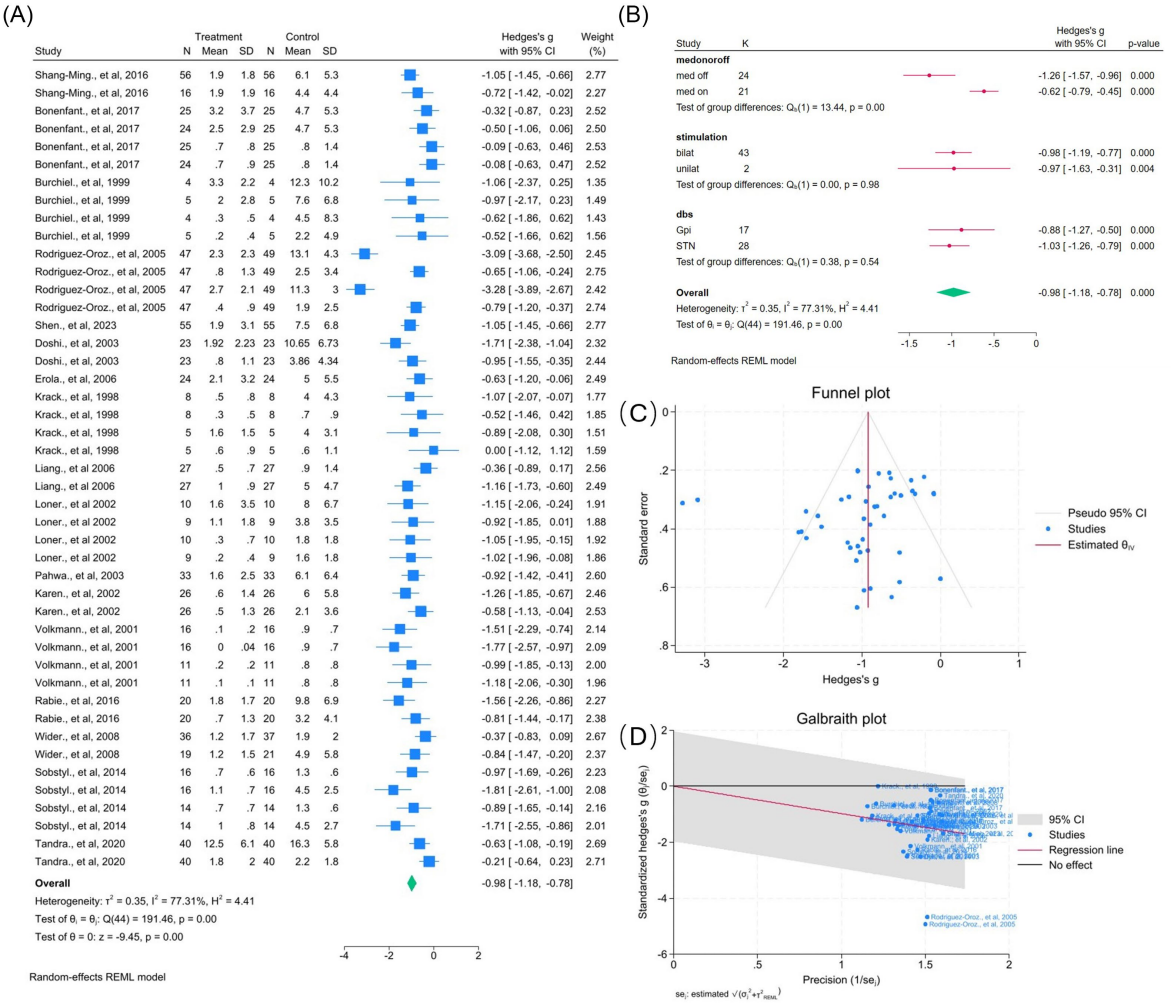
Meta-analysis of the effects of DBS on tremor improvement in PD. **(A)** Forest plot illustrating the overall effect of DBS on tremor subscores derived from the UPDRS-III before and after surgery. **(B)** Subgroup analyses stratified by medication status (*med-on* vs. *med-off*), stimulation type (bilateral vs. unilateral), and stimulation target (GPi vs. STN). **(C)** Funnel plot evaluating potential publication bias with pseudo 95% confidence limits. **(D)** Galbraith plot identifying possible contributors to between-study heterogeneity.

Seventeen studies (45 outcomes) showed a significant tremor reduction (WMD = −0.98, 95% CI –1.18 to −0.78; *p* < 0.05; *I*^2^ = 77.3%). Subgroup analyses demonstrated consistent variability across medication status, laterality, and target (Figure 4B). Funnel asymmetry suggested bias, but Egger’s and Begg’s tests were non-significant (Figure 4C). Removing two outliers reduced heterogeneity (*I*^2^ = 47.5%) and improved symmetry (Figure 4D, Supplementary Figure S2).

#### 3.3.2 Rigidity

**Figure 5:**
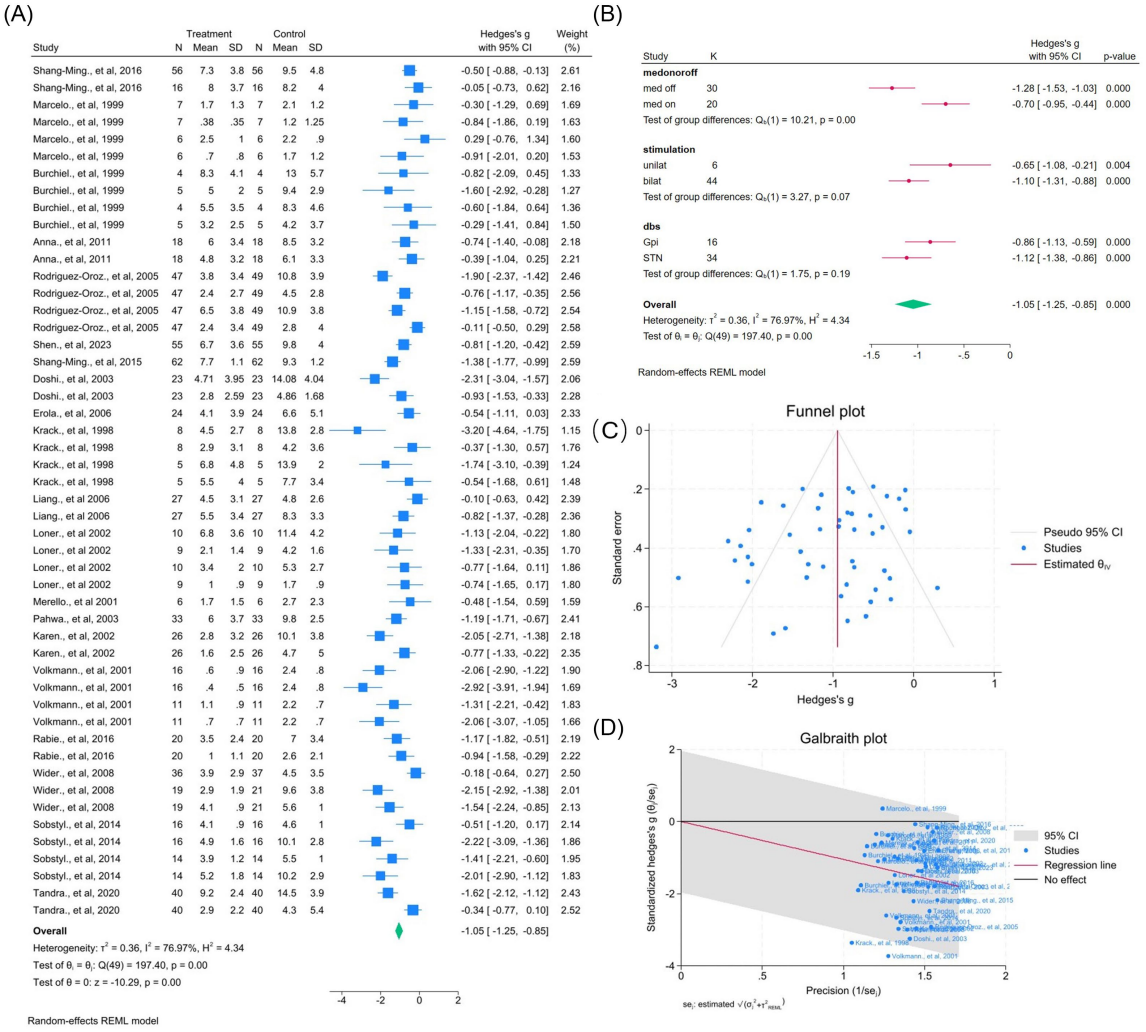
Meta-analysis of the effects of DBS on rigidity improvement in PD. **(A)** Meta-analysis of the effect of deep brain stimulation on rigidity scores. **(A)** Forest plot illustrating the overall effect of DBS on rigidity subscores from the UPDRS-III before and after surgery. **(B)** Subgroup analyses stratified by medication status (med-on vs. med-off), stimulation type (bilateral vs. unilateral), and stimulation target (GPi vs. STN). **(C)** Funnel plot assessing potential publication bias using pseudo 95% confidence limits. **(D)** Galbraith plot identifying possible contributors to between-study heterogeneity.

Twenty studies (50 outcomes) confirmed significant improvement (WMD = −1.05, 95% CI –1.25 to −0.85; *p* < 0.05; *I*^2^ = 76.9%). Subgroup analyses indicated differences by medication status, laterality, and target (all *p* < 0.0001; Figure 5B). Publication bias was visually present but not statistically confirmed (Figure 5C). Excluding two outliers slightly improved heterogeneity (*I*^2^ = 73.5%; Figure 5D, Supplementary Figure S3).

#### 3.3.3 Akinesia

**Figure 6:**
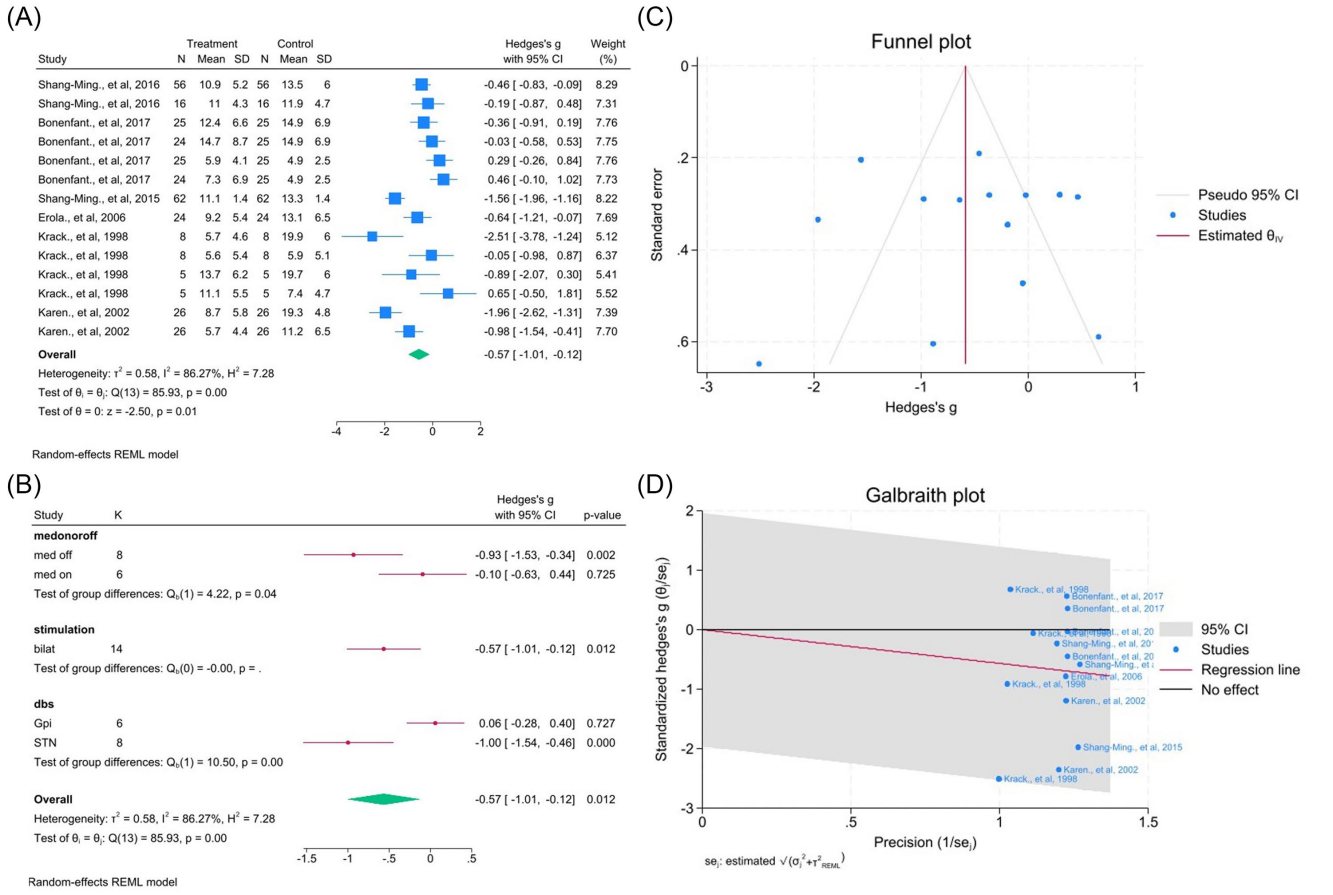
Meta-analysis of the effects of DBS on akinesia improvement in PD. **(A)** Forest plot illustrating the overall effect of DBS on akinesia subscores from the UPDRS-III before and after surgery. **(B)** Subgroup analyses stratified by medication status (*med-on* vs. *med-off*), stimulation type (bilateral vs. unilateral), and stimulation target (GPi vs. STN). **(C)** Funnel plot evaluating potential publication bias using pseudo 95% confidence limits. **(D)** Galbraith plot identifying possible contributors to between-study heterogeneity.

Six studies (14 outcomes) showed significant improvement (WMD = −0.57, 95% CI –1.01 to −0.12; *p* < 0.05; *I*^2^ = 86.3%; Figure 6A). Subgroup results differed across medication and target factors (all *p* < 0.05; Figure 6B). No bias was detected (Figure 6C). All studies lay within the 95% CI range (Figure 6D).

#### 3.3.4 Bradykinesia

**Figure 7:**
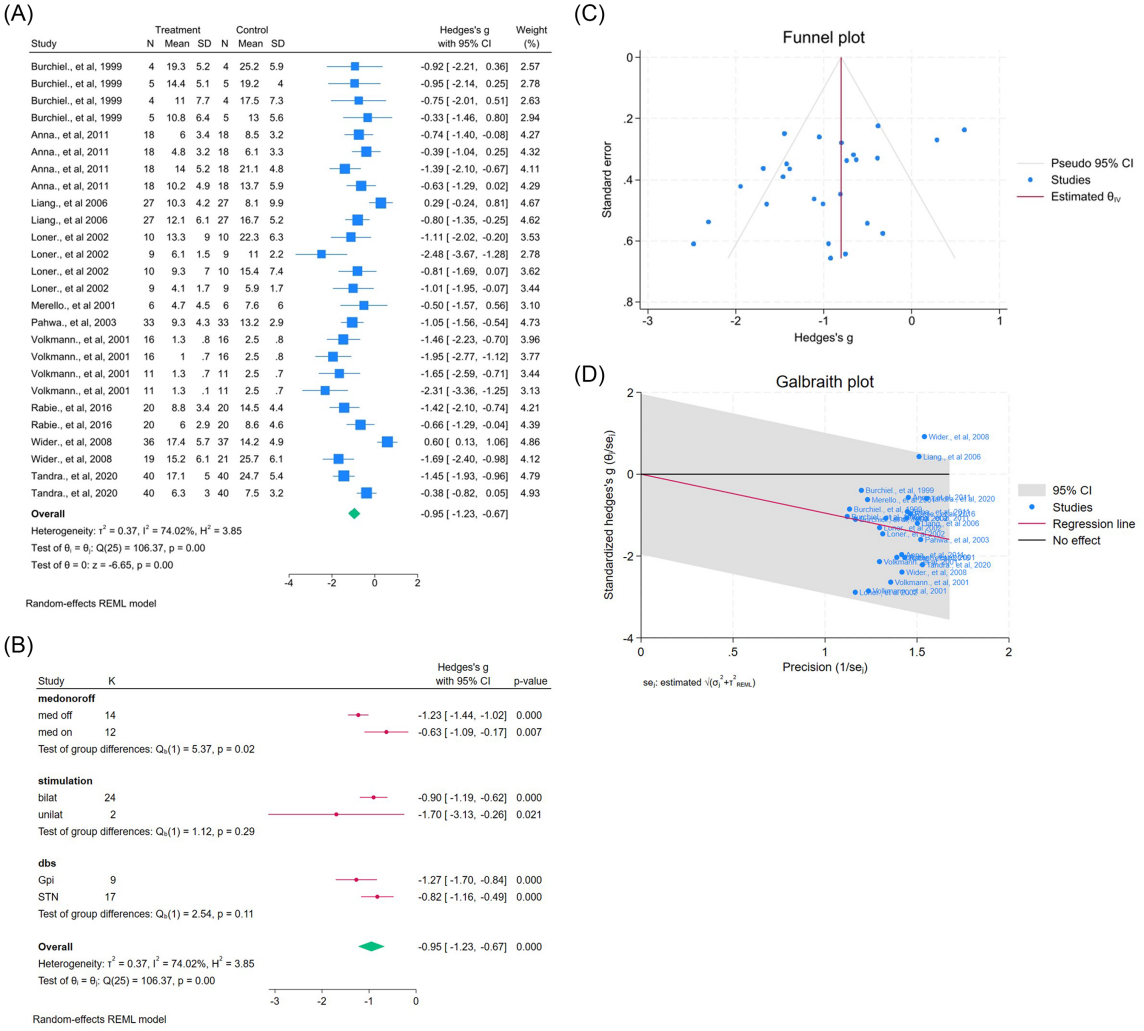
Meta-analysis of the effects of DBS on bradykinesia improvement in PD. **(A)** Forest plot showing the pooled effect of DBS on bradykinesia subscores from the UPDRS-III before and after surgery. **(B)** Subgroup analyses by medication status (*med-on* vs. *med-off*), stimulation type (bilateral vs. unilateral), and target site (GPi vs. STN). **(C)** Funnel plot assessing potential publication bias using pseudo 95% confidence limits. **(D)** Galbraith plot visualizing the sources of between-study heterogeneity.

Ten studies (26 outcomes) demonstrated improvement (WMD = −0.95, 95% CI –1.23 to −0.67; *p* < 0.05; *I*^2^ = 74.0%; Figure 7A). Subgroup differences were noted for medication (*p* = 0.02), laterality (*p* = 0.29), and target (*p* = 0.11) (Figure 7B). Egger’s but not Begg’s test suggested bias (Figure 7C). Excluding one outlier reduced heterogeneity (*I*^2^ = 63.9%) and improved symmetry (Figure 7D, Supplementary Figure S4).

### 3.4 Effects on dyskinesia and UPDRS Part IV

**Figure 8:**
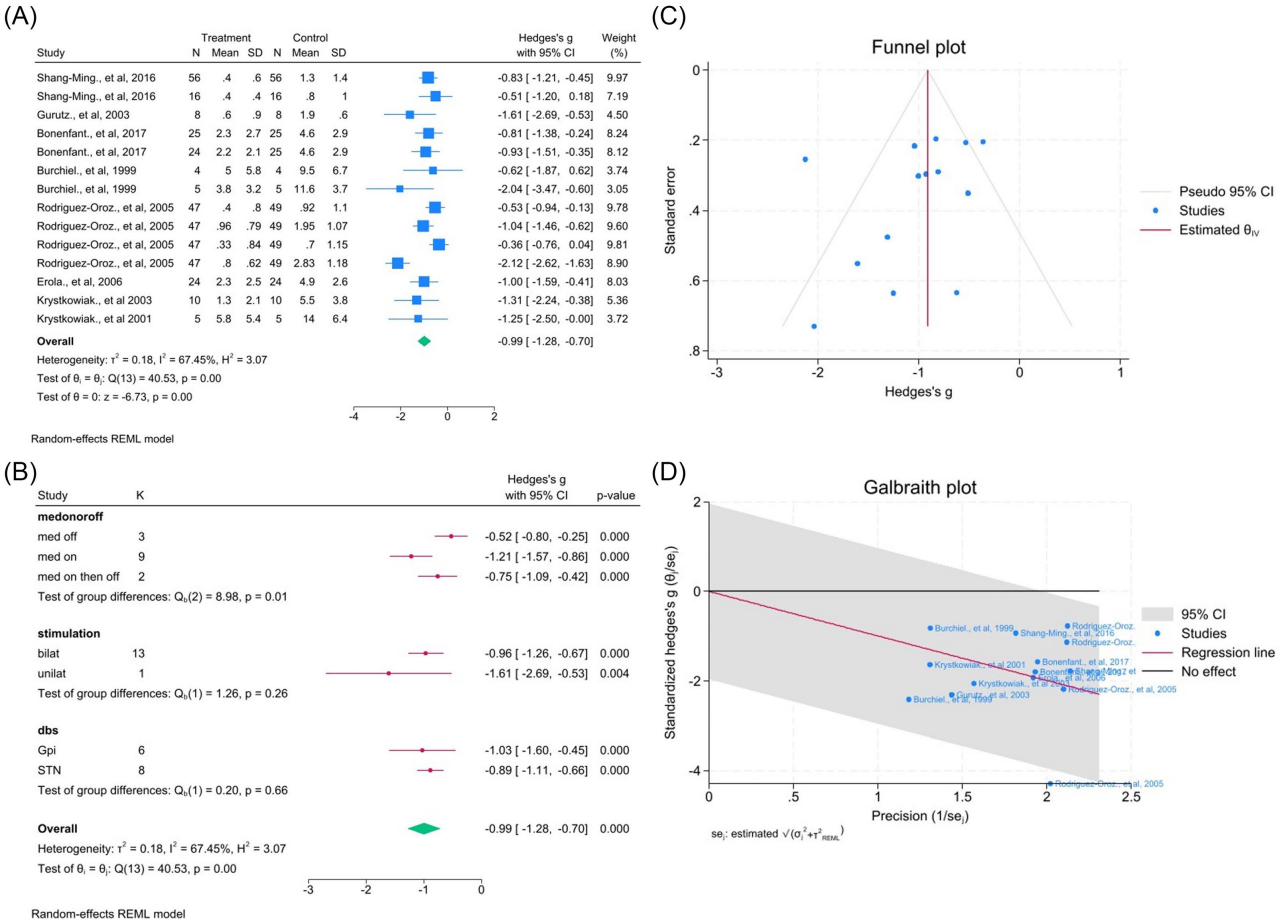
Meta-analysis of the effects of DBS on dyskinesia in PD. **(A)** Forest plot showing the pooled effect of DBS on dyskinesia scores before and after surgery. **(B)** Subgroup analyses stratified by medication status (med-on vs. med-off), stimulation type (bilateral vs. unilateral), and target site (GPi vs. STN). **(C)** Funnel plot assessing potential publication bias using pseudo 95% confidence limits. **(D)** Galbraith plot visualizing potential sources of between-study heterogeneity.

**Figure 9:**
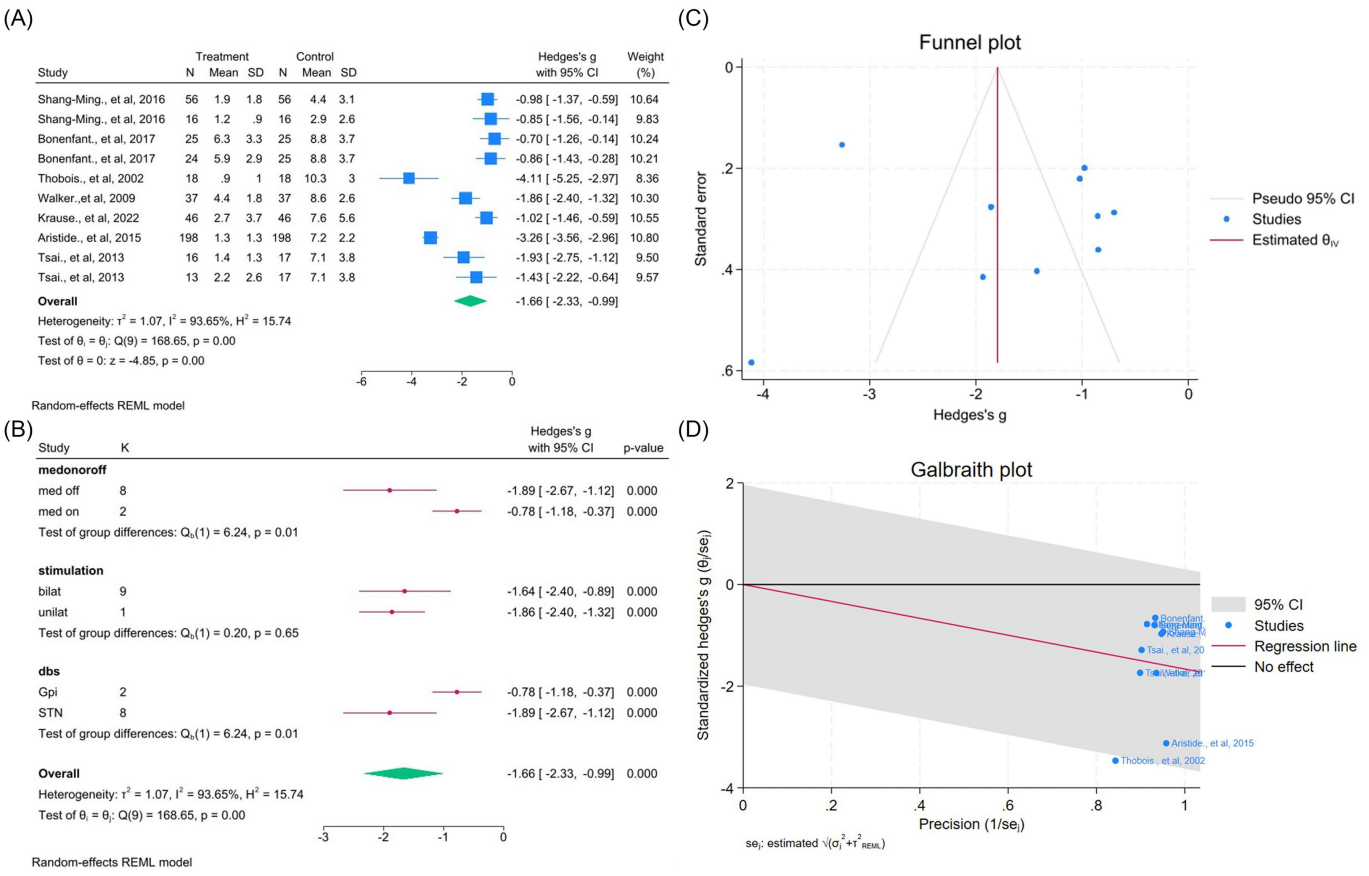
Meta-analysis of the effects of DBS on Unified Parkinson’s Disease Rating Scale (UPDRS) Part IV (motor complications) in PD. **(A)** Forest plot illustrating the pooled effect of DBS on UPDRS Part IV scores. **(B)** Subgroup analyses stratified by medication status (med-on vs. med-off), stimulation type (bilateral vs. unilateral), and target site (GPi vs. STN). **(C)** Funnel plot evaluating potential publication bias with pseudo 95% confidence limits. **(D)** Galbraith plot identifying possible contributors to between-study heterogeneity.

DBS significantly improved dyskinesia across eight studies (14 outcomes; WMD = −0.99, 95% CI –1.28 to −0.70; *p* < 0.05; *I*^2^ = 67.5%; Figures 8A–D, Supplementary Figure S5). Similarly, UPDRS Part IV improved in seven studies (10 outcomes; WMD = −1.66, 95% CI –2.33 to −0.99; *p* < 0.05; *I*^2^ = 93.6%; Figures 9A–D, Supplementary Figure S6), indicating reduction of motor complications.

### 3.5 Effects on gait and axial symptoms

#### 3.5.1 Gait

**Figure 10:**
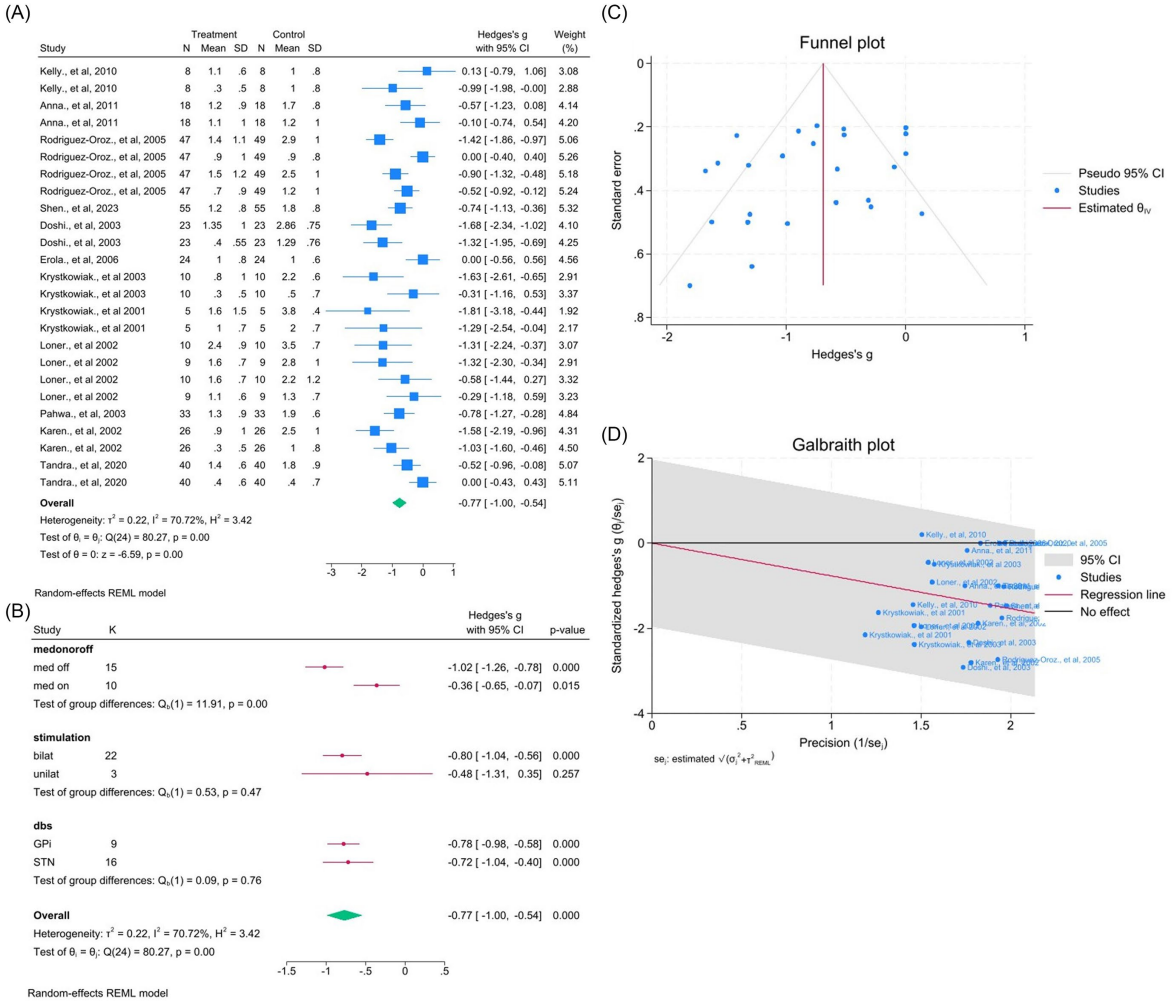
Meta-analysis of the effects of DBS on gait performance in PD. **(A)** Forest plot showing the pooled effect of DBS on UPDRS-III gait subscores before and after surgery. **(B)** Subgroup analyses by medication status (med-on vs. med-off), stimulation type (bilateral vs. unilateral), and target site (GPi vs. STN). **(C)** Funnel plot assessing potential publication bias using pseudo 95% confidence limits. **(D)** Galbraith plot visualizing sources of between-study heterogeneity.

Twelve studies (25 outcomes) showed significant improvement (WMD = −0.77, 95% CI –1.00 to −0.54; *p* < 0.05; *I*^2^ = 70.7%; Figures 10A–D). Egger’s and Begg’s tests were not significant despite visual asymmetry.

#### 3.5.2 Axial symptoms

**Figure 11:**
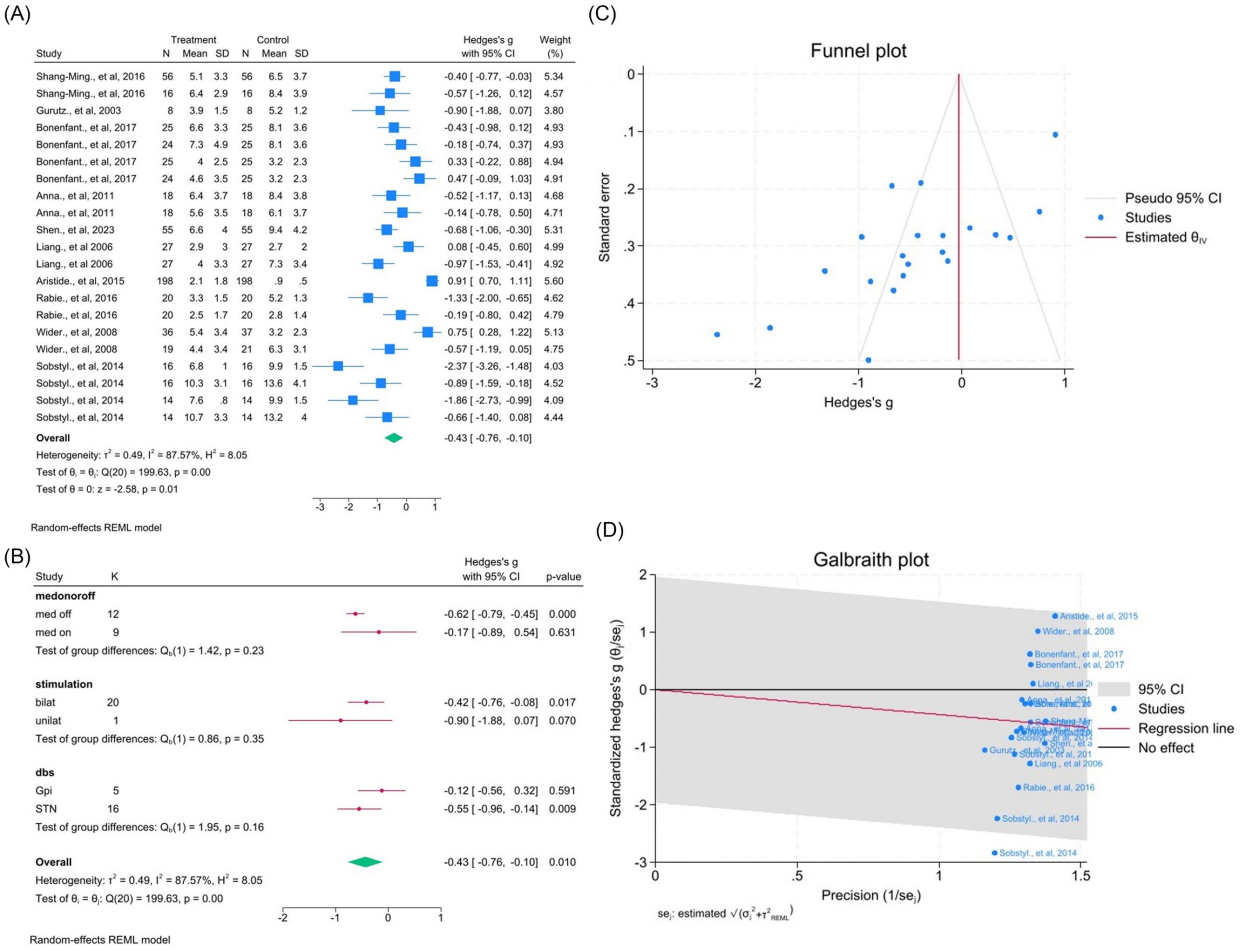
Meta-analysis of the effects of DBS on axial symptoms in Pd. **(A)** Forest plot showing the pooled effect of DBS on axial subscores from the UPDRS-III before and after surgery. **(B)** Subgroup analyses stratified by medication status (med-on vs. med-off), stimulation type (bilateral vs. unilateral), and target site (GPi vs. STN). **(C)** Funnel plot assessing potential publication bias using pseudo 95% confidence limits. **(D)** Galbraith plot visualizing potential sources of between-study heterogeneity.

Ten studies (21 outcomes) demonstrated moderate improvement (WMD = −0.43, 95% CI –0.76 to −0.10; *p* < 0.05; *I*^2^ = 87.6%; Figures 11A–D, Supplementary Figure S7). Removing one outlier slightly reduced heterogeneity (*I*^2^ = 84.6%).

### 3.6 Effects on speech

**Figure 12:**
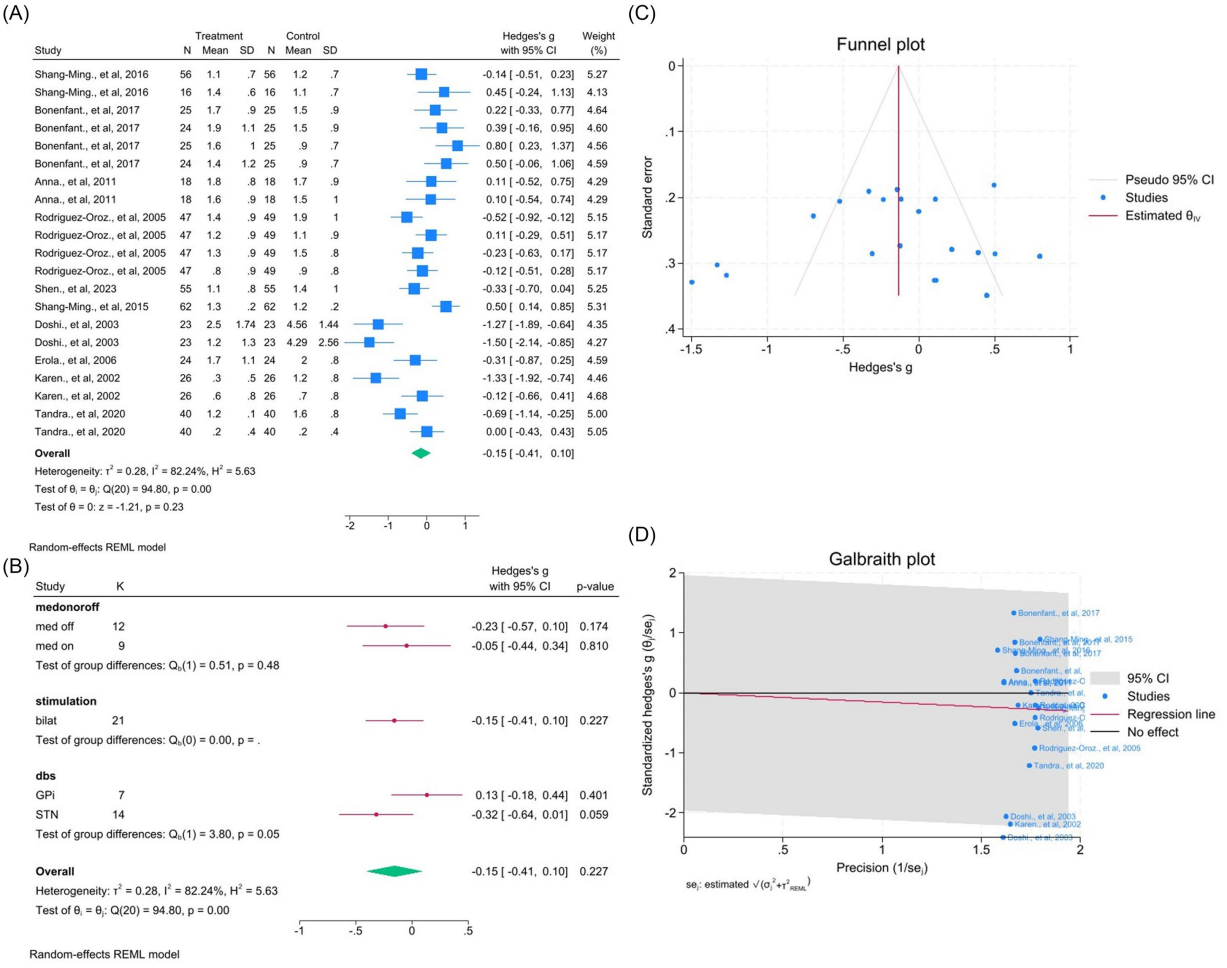
Meta-analysis of the effects of DBS on speech outcomes in PD. **(A)** Forest plot summarizing the overall effect of DBS on UPDRS-III speech subscores before and after surgery. **(B)** Subgroup analyses by medication status (med-on vs. med-off), stimulation type (bilateral vs. unilateral), and stimulation target (GPi vs. STN). **(C)** Funnel plot assessing publication bias using pseudo 95% confidence intervals. **(D)** Galbraith plot identifying potential sources of between-study heterogeneity.

Ten studies (21 outcomes) showed no significant effect on speech (WMD = −0.15, 95% CI –0.41 to 0.10; *p* > 0.05; *I*^2^ = 82.2%; Figures 12A–D, Supplementary Figure S8). Subgroup results showed no difference across medication, laterality, or target.

### 3.7 Effects on UPDRS Parts I and II

**Figure 13:**
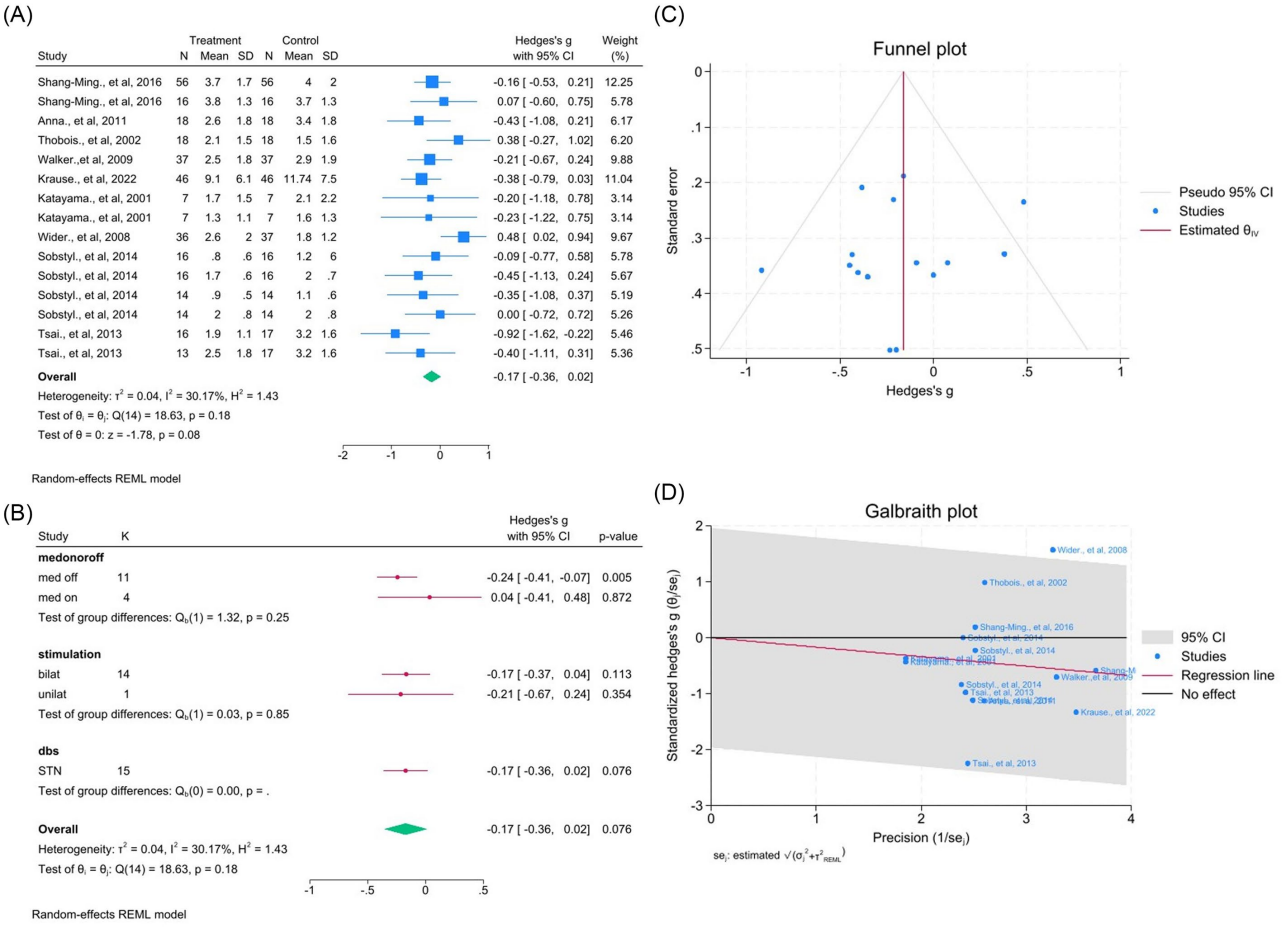
Meta-analysis of the effects of DBS on UPDRS Part I (non-motor experiences of daily living). **(A)** Forest plot showing the pooled effects of DBS on UPDRS Part I scores. **(B)** Subgroup analyses by medication status (med-on vs. med-off), stimulation type (bilateral vs. unilateral), and target site (GPi vs. STN). **(C)** Funnel plot evaluating publication bias with pseudo 95% confidence limits. **(D)** Galbraith plot identifying possible contributors to heterogeneity.

**Figure 14:**
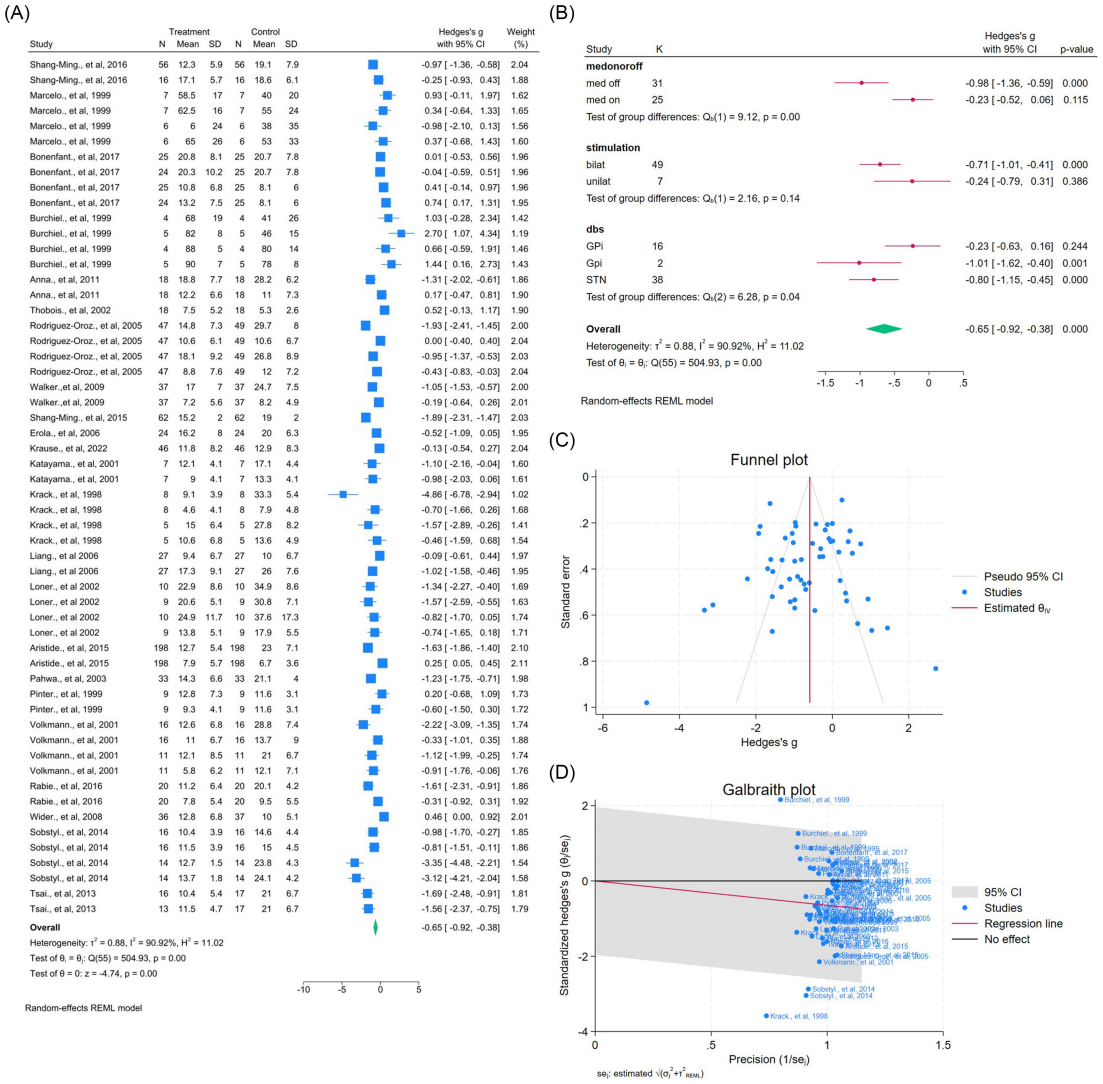
Meta-analysis of the effects of DBS on UPDRS Part II (motor experiences of daily living). **(A)** Forest plot illustrating pooled DBS effects on UPDRS Part II scores before and after surgery. **(B)** Subgroup analyses stratified by medication status, stimulation type, and stimulation target. **(C)** Funnel plot assessing potential publication bias using pseudo 95% confidence limits. **(D)** Galbraith plot visualizing possible sources of heterogeneity.

Nine studies (15 outcomes) revealed a slight but nonsignificant change in Part I (WMD = −0.17, 95% CI –0.36 to 0.02; *p* > 0.05; *I*^2^ = 30.7%; Figures 13A–D, Supplementary Figure S9), which became significant after outlier removal (*p* < 0.05). Twenty-three studies (56 outcomes) demonstrated significant Part II improvement (WMD = −0.65, 95% CI –0.92 to −0.38; *p* < 0.05; *I*^2^ = 90.9%; Figures 14A–D, Supplementary Figure S10).

### 3.8 Effects on UPDRS total score

**Figure 15:**
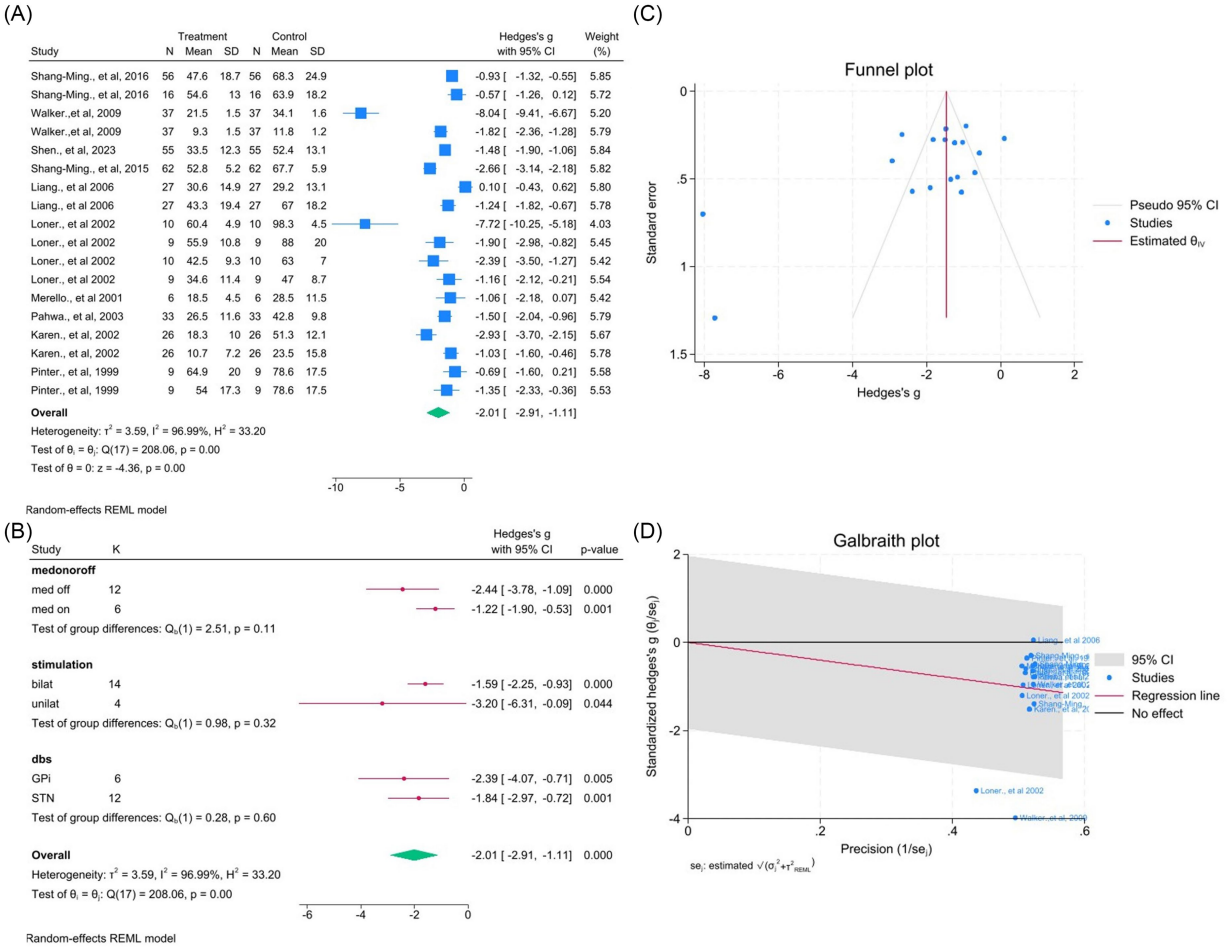
Meta-analysis of the effects of DBS on total UPDRS scores. **(A)** Forest plot summarizing the overall change in UPDRS total scores following DBS. **(B)** Subgroup analyses by medication status, stimulation type, and target site. **(C)** Funnel plot assessing potential publication bias using pseudo 95% confidence limits. **(D)** Galbraith plot displaying potential contributors to heterogeneity.

Ten studies (18 outcomes) showed a robust overall improvement in total UPDRS scores (WMD = −2.01, 95% CI –2.91 to −1.11; *p* < 0.05; *I*^2^ = 96.9%; Figures 15A–D, Supplementary Figure S11), confirming the global therapeutic benefit of DBS.

## 4 Discussion

DBS has become a crucial therapeutic intervention for PD, effectively addressing motor complications that substantially affect patients’ quality of life (Deuschl et al., 2013). This meta-analysis systematically evaluated the effects of DBS on various motor symptoms in PD, encompassing tremor, rigidity, bradykinesia, axial symptoms, dyskinesia, and speech function. The pooled results demonstrated a significant improvement in overall motor function, as evidenced by reductions in UPDRS-III scores, with consistent benefits observed for tremor, rigidity, akinesia, bradykinesia, and dyskinesia. However, speech function and Part I scores did not show significant improvement, and some studies even reported deterioration post-DBS. Subgroup analyses revealed that the effectiveness of DBS varied depending on the stimulation target (STN vs. GPi), stimulation type (bilateral vs. unilateral), and medication status (med on vs. med off). Despite these clear benefits, heterogeneity remained substantial across studies, suggesting that additional factors may contribute to variations in DBS outcomes.

DBS exerts its therapeutic effects by modulating pathological neural activity within the basal ganglia-thalamocortical circuit, which becomes dysregulated due to dopaminergic neuronal loss in PD. The principal DBS targets are the STN and GPi, each influencing motor function through distinct mechanisms. Tremor, rigidity, bradykinesia, akinesia, dyskinesia, axial symptoms, and speech dysfunction are key motor complications in PD that DBS aims to alleviate by modulating dysfunctional basal ganglia circuits (Davidson et al., 2024). Tremor in PD is associated with abnormal oscillatory activity within the cortico-thalamic-basal ganglia loop (Timmermann et al., 2002), and STN-DBS has been shown to desynchronize excessive neuronal synchronization, thereby reducing tremor amplitude and frequency (Daneshzand et al., 2018). While GPi-DBS is less commonly used for tremor management, it may still suppress tremor through modulation of thalamocortical output (Muralidharan et al., 2017). In contrast, rigidity and bradykinesia result from excessive inhibitory outflow from the GPi, leading to suppressed movement initiation and execution (Chou and Hurtig, 2012). STN-DBS reduces hyperactivity within the STN, indirectly decreasing excessive GPi inhibition and facilitating improved motor function, while GPi-DBS directly reduces abnormal inhibitory signaling, thereby restoring thalamocortical excitability and enhancing movement fluidity. The observed improvements in rigidity and bradykinesia in this analysis are consistent with previous findings demonstrating that both STN and GPi stimulation effectively reduce motor stiffness and movement slowness.

In addition, LID, which arises from abnormal striatal dopamine signaling and altered synaptic plasticity (Brotchie et al., 2005), was significantly reduced in this meta-analysis. GPi-DBS, in particular, has been shown to attenuate dyskinesia severity by directly modulating aberrant inhibitory outflow from the GPi, thereby normalizing cortical excitability, whereas STN-DBS may indirectly alleviate dyskinesia by enabling reductions in levodopa dosage, subsequently lowering the risk of drug-induced motor fluctuations (Stefani et al., 2019). In addition, both STN-DBS and GPi-DBS lead to significant improvements in overall motor function, including Akinesia and gait performance. In this meta-analysis, the results demonstrated that these function showed a statistically significant improvement following DBS. Both STN-DBS and GPi-DBS contributed to enhanced mobility, including improvements in stride length, walking speed, and movement initiation. However, individual variability exists, with some studies suggesting that postural instability may persist or worsen over time, particularly in certain patients receiving long-term STN stimulation. This effect may be attributed to unintended modulation of brainstem locomotor networks, which play a key role in balance and gait coordination (Camicioli and Nutt, 2007). Despite these variations, the overall findings confirm that DBS significantly improves gait-related mobility in PD patients.

Furthermore, comparative analyses between GPi and STN stimulation have revealed distinct clinical advantages associated with each target. STN-DBS is often favored for patients requiring substantial reduction in dopaminergic medication, as stimulation of the STN allows significant decreases in levodopa dosage without compromising motor control, thereby reducing long-term medication-induced complications. Conversely, GPi-DBS is generally superior in controlling levodopa-induced dyskinesia, providing robust suppression of involuntary movements even when medication dosage remains unchanged (Liu et al., 2019). A meta-analysis in 2025 indicates Verbal fluency often declined regardless of target, while working memory effects were mixed, slightly favoring GPi stimulation in one study (Trotman et al., 2025). Although evidence does not clearly demonstrate that STN stimulation provides superior tremor control compared to GPi-DBS, it remains the more commonly selected target in clinical practice (Trotman et al., 2025). Furthermore, GPi targeting is often recommended for patients exhibiting, or at high risk of developing, cognitive impairments or psychiatric disturbances (Follett et al., 2010; Odekerken et al., 2015). It may also be advantageous for individuals experiencing moderate swallowing difficulties or gait abnormalities (Rughani et al., 2018).

Speech impairment in PD remains a particularly complex motor symptom, involving cortical motor areas, the basal ganglia, and the cerebellum (Brabenec et al., 2017). The results in this meta-analysis indicate that DBS does not significantly improve speech function, and in some cases, it may even lead to deterioration. This aligns with prior reports suggesting that STN-DBS can impair articulatory precision and phonation, potentially due to spillover stimulation affecting nearby corticobulbar pathways (Tabari et al., 2024). These findings show the importance of precise electrode placement and stimulation parameter adjustments to minimize adverse speech-related effects while optimizing therapeutic outcomes. From a clinical perspective, speech deterioration after DBS represents a critical challenge for patient quality of life, even when motor symptoms are substantially improved. Careful preoperative planning to identify individualized targets, intraoperative microelectrode recording to refine trajectory, and postoperative programming that balances motor benefit with minimal spread to adjacent fibers are essential. Adjusting stimulation amplitude, pulse width, or frequency, as well as employing directional leads and current steering technologies, can help limit current diffusion to speech-related neural pathways. Moreover, multidisciplinary management, including speech therapy and postoperative monitoring, is recommended to mitigate functional decline. These measures can assist clinicians in maintaining optimal therapeutic efficacy while reducing DBS-induced speech impairments.

In addition to UPDRS-III, we analyzed axial symptoms, UPDRS Part I, Part II, Part IV, and UPDRS-Total to comprehensively assess DBS outcomes. Axial symptoms, including postural instability and freezing of gait, showed significant improvement, suggesting DBS may enhance gait and balance control, though variability remains. UPDRS Part I, which initially showed no significant change, became significant after removing sources of heterogeneity, indicating potential benefits for non-motor symptoms in select patients. UPDRS Part II showed partial improvement, reflecting enhanced gross motor function, though fine motor control and speech limitations may still affect daily life. UPDRS Part IV also significantly improved, reinforcing its role in stabilizing motor fluctuations and reducing dyskinesia. In all, the significant overall reduction in UPDRS-Total scores highlights a broad motor benefit.

Despite the strong overall effect of DBS on motor function, substantial heterogeneity was observed across studies, with *I*^2^ values remaining high in certain analyses even after removing outliers, while some analyses showed a reduction in heterogeneity. This suggests that variability arises from multiple interrelated factors rather than isolated study design differences. Variability in study design, including differences in follow-up duration, patient selection criteria, and baseline disease severity, likely contributed to the inconsistent findings, as DBS efficacy can be influenced by disease stage and progression (Mahlknecht et al., 2022). Additionally, stimulation parameters were not standardized across studies, with differences in electrode placement, stimulation intensity, and programming settings affecting treatment outcomes (Jakobs et al., 2020). Beyond these methodological differences, disease progression itself may contribute to persistent heterogeneity, particularly in axial symptoms and speech dysfunction, which tend to worsen over time due to non-dopaminergic neurodegeneration rather than DBS-related effects. The variability in gait and speech outcomes further complicates interpretation, as these symptoms are influenced by patient-specific factors, DBS target selection, and potential unintended stimulation of adjacent structures. Interestingly, even after excluding studies identified as heterogeneity sources in the Galbraith plot, some analyses showed only minimal reductions in *I*^2^ values, indicating that intrinsic patient variability, unmeasured confounders, or long-term neural adaptations to DBS may also contribute to outcome differences.

Given these complexities, future research should focus on methodological standardization, longer-term follow-up, and refined patient stratification to improve the comparability and reproducibility of DBS outcomes. Advances in neuroimaging, such as diffusion tractography and connectome-based targeting, may enhance electrode placement accuracy and enable more individualized stimulation strategies. In addition, the development of adaptive or closed-loop DBS systems holds promise for dynamically adjusting stimulation parameters in response to real-time neural signals. Exploration of non-traditional targets, such as the pedunculopontine nucleus for gait and postural control, could further expand the therapeutic scope of DBS. Collectively, these innovations may optimize clinical outcomes, reduce adverse effects, and refine patient selection to achieve more personalized and effective neuromodulation for Parkinson’s disease.

## 5 Conclusion

This study reaffirms the clinical effectiveness of deep brain stimulation (DBS) in alleviating the cardinal motor symptoms of Parkinson’s disease, including tremor, rigidity, bradykinesia, and dyskinesia. Subgroup analyses highlight that treatment response is influenced by stimulation target (STN vs. GPi), laterality, and medication status. However, outcomes related to gait and speech remain inconsistent, with some evidence indicating limited or even adverse effects on speech performance. These findings underscore that while DBS provides robust motor benefits, its effects on axial and communicative functions are less predictable. Future refinements in DBS technology and programming should prioritize these challenging domains and integrate individualized stimulation strategies to maximize clinical benefit and quality of life in patients with PD.

## Data Availability

The original contributions presented in the study are included in the article/Supplementary material, further inquiries can be directed to the corresponding author.
