# Supplementary material for: Effect of deep brain stimulation on motor complications in Parkinson’s disease: a systematic review and meta-analysis
Source: Front Hum Neurosci. 2025 Dec 18;19:1684229. doi: 10.3389/fnhum.2025.1684229 (PMC12756497; doi:10.3389/fnhum.2025.1684229)
Supplement: Supplementary file 1 [file Data_Sheet_1.docx]

**Supporting Information**

**1.** **Meta-analysis of Deep Brain Stimulation's Effects on UPDRS-III Score**

two studies (Loner. et al. 2002 and Volkmann., et al, 2001) fall outside the confidence interval, suggesting they may contribute to the observed heterogeneity. The overall heterogeneity did not significantly decrease after eliminating these two studies (I^2^ = 85.81%), suggesting that different researchers were still reporting contradictory findings. (Figure S1A). The results of subgroup analyses are shown in Figure S1B. The funnel plot (Figure S1C) showed improved symmetry, suggesting a reduction in publication bias after excluding the outliers. Therefore, although the exclusion of studies outside the confidence interval slightly improved the model fit, substantial heterogeneity remains, necessitating further investigation into the sources of variability.


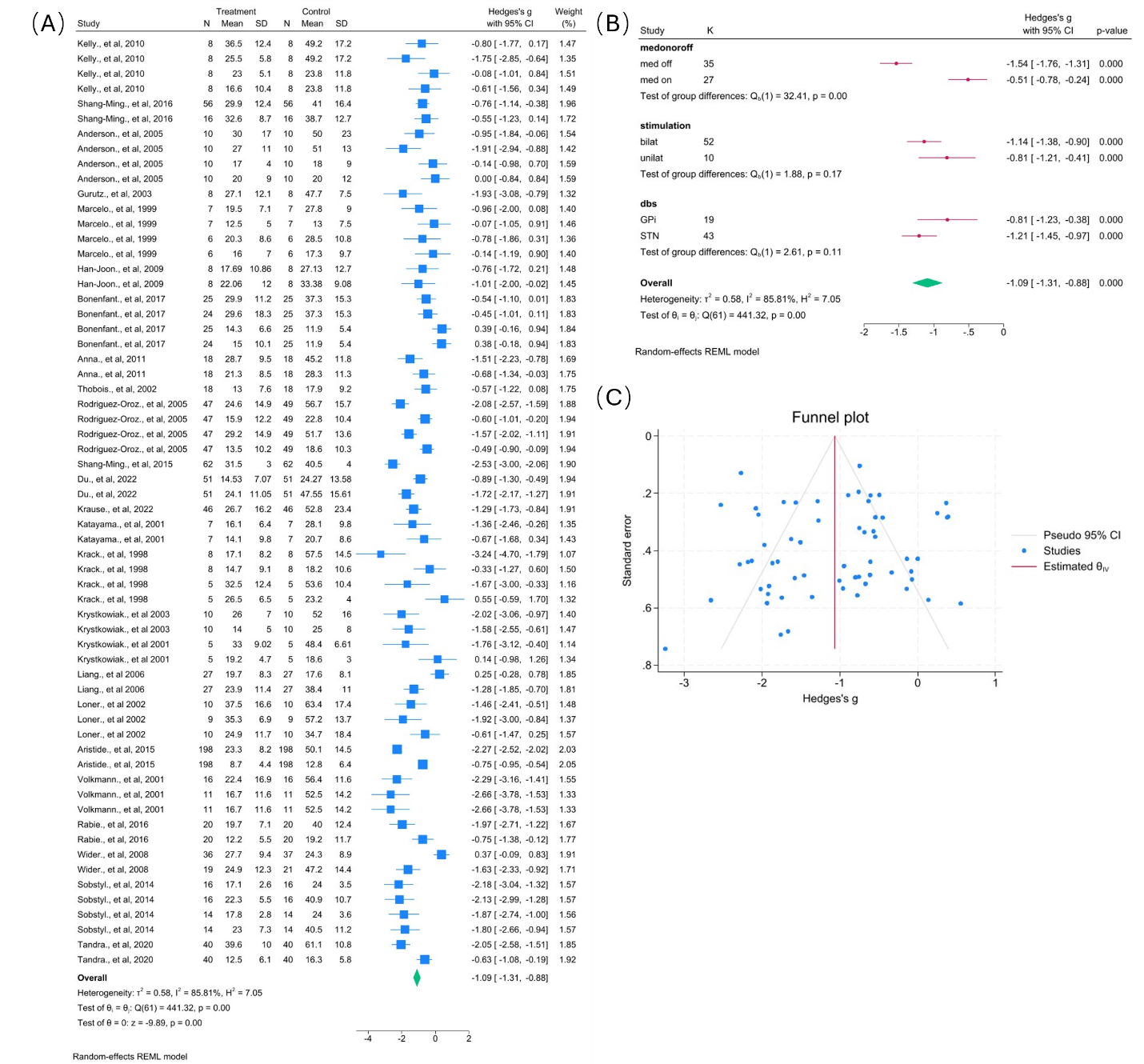


**Figure S1.** Meta-analysis of the effect of Deep Brain Stimulation on UPDRS-III after removing sources of heterogeneity. (A) Meta-analysis of the effect of Deep Brain Stimulation on UPDRS-III scores. (B) Subgroup analysis by medication status (med on vs. med off), stimulation type (bilateral vs. unilateral), and target site (GPi vs. STN). (C) Funnel plot assessing publication bias after removing sources of heterogeneity, with pseudo 95% CI. Diamonds represent the pooled effect estimate for overall and subgroup analyses. Data are represented as Hedges's g with 95% CI, using the random-effects REML model. Between-study heterogeneity is quantified by I^2^, with significance at p < 0.05. Test of homogeneity (θ_i_ = θ_j_) of study-specific effect sizes, with the chi-squared test statistic rejected if p < 0.05.

**2.** **Meta-analysis of Deep Brain Stimulation's Effects on Tremor**

After removing two results from one study (Rodringuez-Oroz. et al., 2005), the overall heterogeneity decreased to a moderate level (I^2^ = 47.45%), suggesting reduced variability among the included studies (Figure S2A). The results of subgroup analyses are shown in Figure S2B. The funnel plot (Figure S2C) showed substantial improved symmetry, indicating a reduction in publication bias after excluding the outliers.


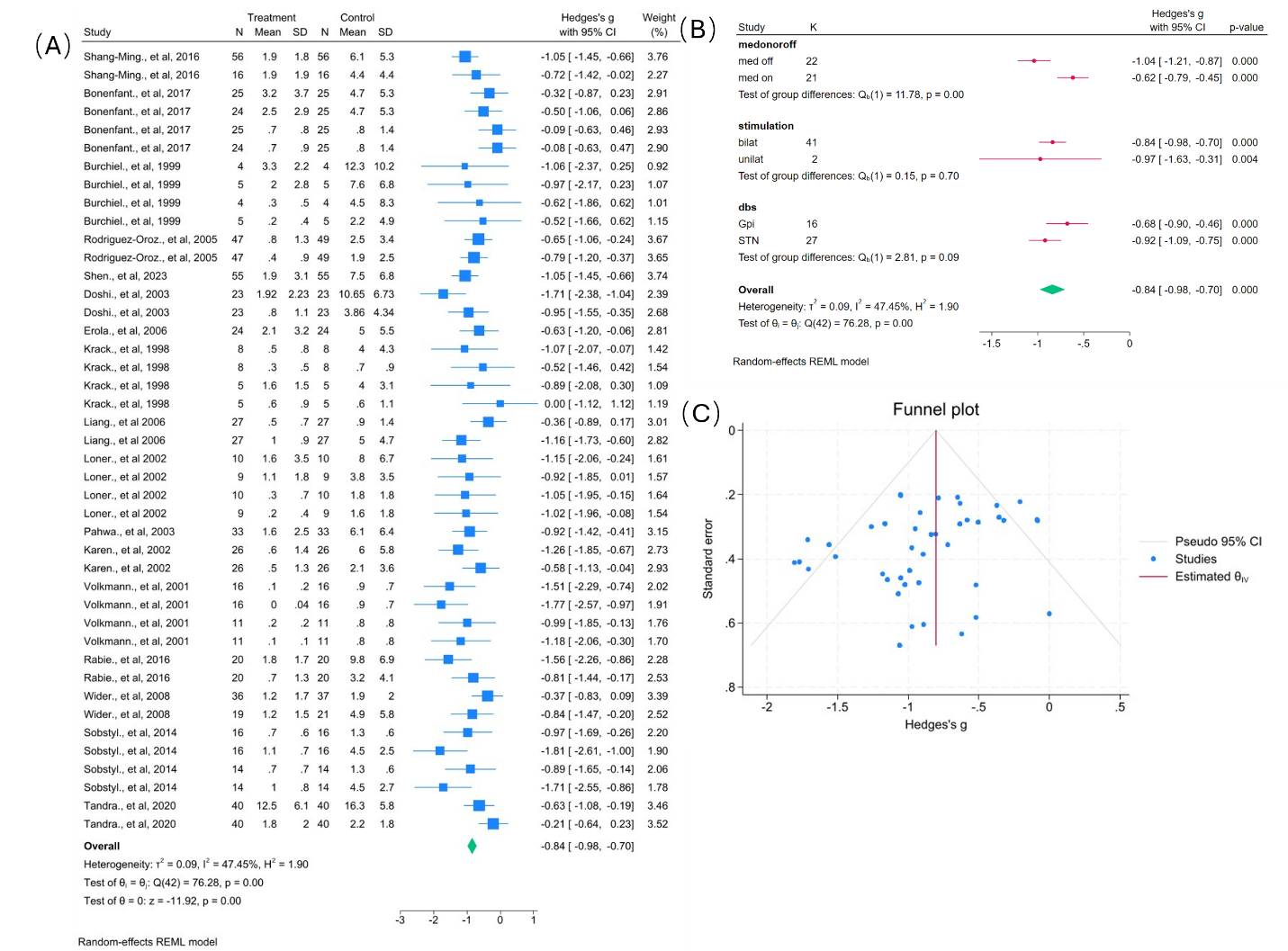


**Figure S2.** Meta-analysis of the effect of Deep Brain Stimulation on Tremor after removing sources of heterogeneity. (A) Meta-analysis of the effect of Deep Brain Stimulation on Tremor scores. (B) Subgroup analysis by medication status (med on vs. med off), stimulation type (bilateral vs. unilateral), and target site (GPi vs. STN). (C) Funnel plot assessing publication bias after removing sources of heterogeneity, with pseudo 95% CI. Diamonds represent the pooled effect estimates for overall and subgroup analyses. Data are represented as Hedges's g with 95% CI, using the random-effects REML model. Between-study heterogeneity is quantified by I^2^, with significance at p < 0.05. Test of homogeneity (θ_i_ = θ_j_) of study-specific effect sizes, with the chi-squared test statistic rejected if p < 0.05.

**3.** **Meta-analysis of Deep Brain Stimulation's Effects on Rigidity**

Two studies (Krack. et al, 1998 and Volkmann., et al, 2001) fall outside the confidence interval, suggesting they may contribute to the observed heterogeneity. After excluding these two studies, overall heterogeneity remained largely unchanged (I^2^ = 73.48%), indicating that conflicting results were still reported by different researchers (Figure S3A). The results of subgroup analyses are shown in Figure S3B. The funnel plot (Figure S3C) showed improved symmetry, suggesting a reduction in publication bias after excluding the outliers.


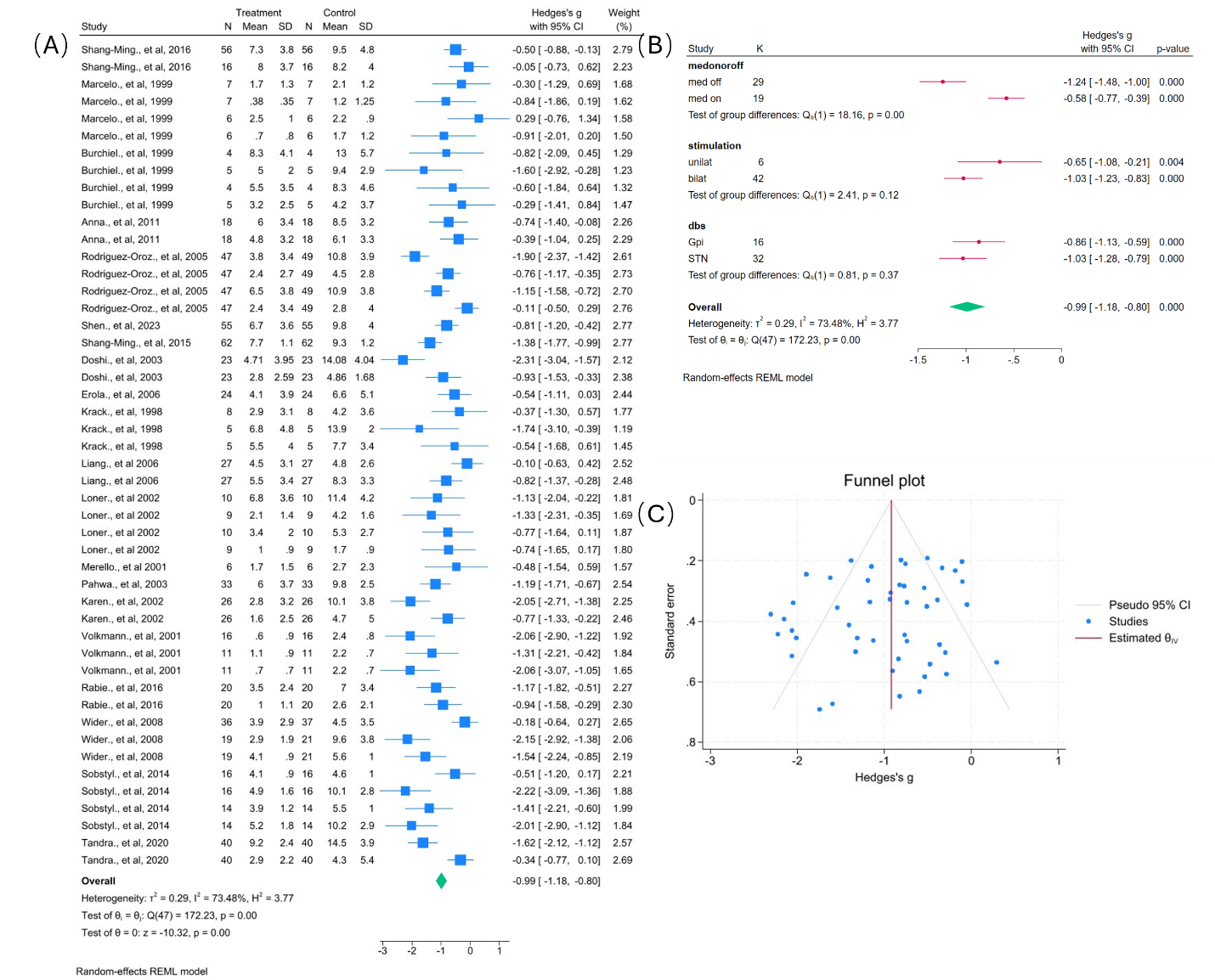


**Figure S3.** Meta-analysis of the effect of Deep Brain Stimulation on Rigidity after removing sources of heterogeneity. (A) Meta-analysis of the effect of Deep Brain Stimulation on Rigidity scores. (B) Subgroup analysis by medication status (med on vs. med off), stimulation type (bilateral vs. unilateral), and target site (GPi vs. STN). (C) Funnel plot assessing publication bias after removing sources of heterogeneity, with pseudo 95% CI. Diamonds represent the pooled effect estimates for overall and subgroup analyses. Data are represented as Hedges's g with 95% CI, using the random-effects REML model. Between-study heterogeneity is quantified by I^2^, with significance at p < 0.05. Test of homogeneity (θ_i_ = θ_j_) of study-specific effect sizes, with the chi-squared test statistic rejected if p < 0.05.

**4.** **Meta-analysis of Deep Brain Stimulation's Effects on Bradykinesia**

One study (Wider., et al, 2008) falls outside the confidence interval, suggesting it may contribute to the observed heterogeneity. After excluding this study, overall heterogeneity remained largely unchanged (I² = 63.95%), indicating persistent discrepancies among researchers' findings (Figure S4A). The results of subgroup analyses are shown in Figure S4B. The funnel plot (Figure S4C) showed improved symmetry, suggesting a reduction in publication bias after excluding the outlier.


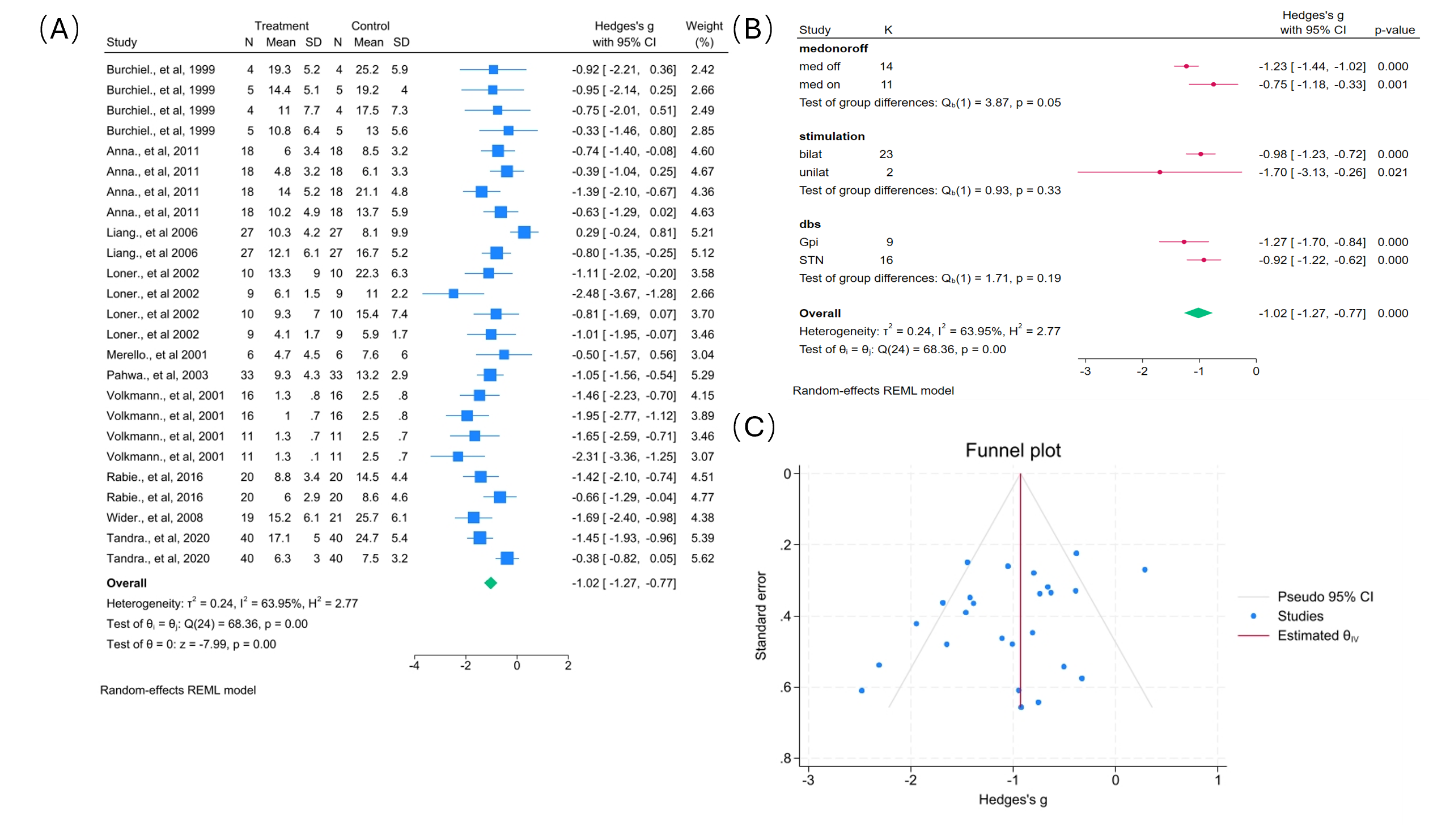


**Figure S4.** Meta-analysis of the effect of Deep Brain Stimulation on Bradykinesia after removing sources of heterogeneity. (A) Meta-analysis of the effect of Deep Brain Stimulation on Bradykinesia scores. (B) Subgroup analysis by medication status (med on vs. med off), stimulation type (bilateral vs. unilateral), and target site (GPi vs. STN). (C) Funnel plot assessing publication bias after removing sources of heterogeneity, with pseudo 95% CI. Diamonds represent the pooled effect estimates for overall and subgroup analyses. Data are represented as Hedges's g with 95% CI, using the random-effects REML model. Between-study heterogeneity is quantified by I^2^, with significance at p < 0.05. Test of homogeneity (θ_i_ = θ_j_) of study-specific effect sizes, with the chi-squared test statistic rejected if p < 0.05.

**5.** **Meta-analysis of Deep Brain Stimulation's Effects on Dyskinesia**

One study falls outside the confidence interval, suggesting it may contribute to the observed heterogeneity. After removing this study, the overall heterogeneity decreased to a low level (I^2^ = 22.81%), suggesting reduced variability among the included studies (Figure S6A). The results of subgroup analyses are shown in Figure S6B. The funnel plot (Figure S6C) showed improved symmetry, showing a reduction in publication bias after excluding the outlier.


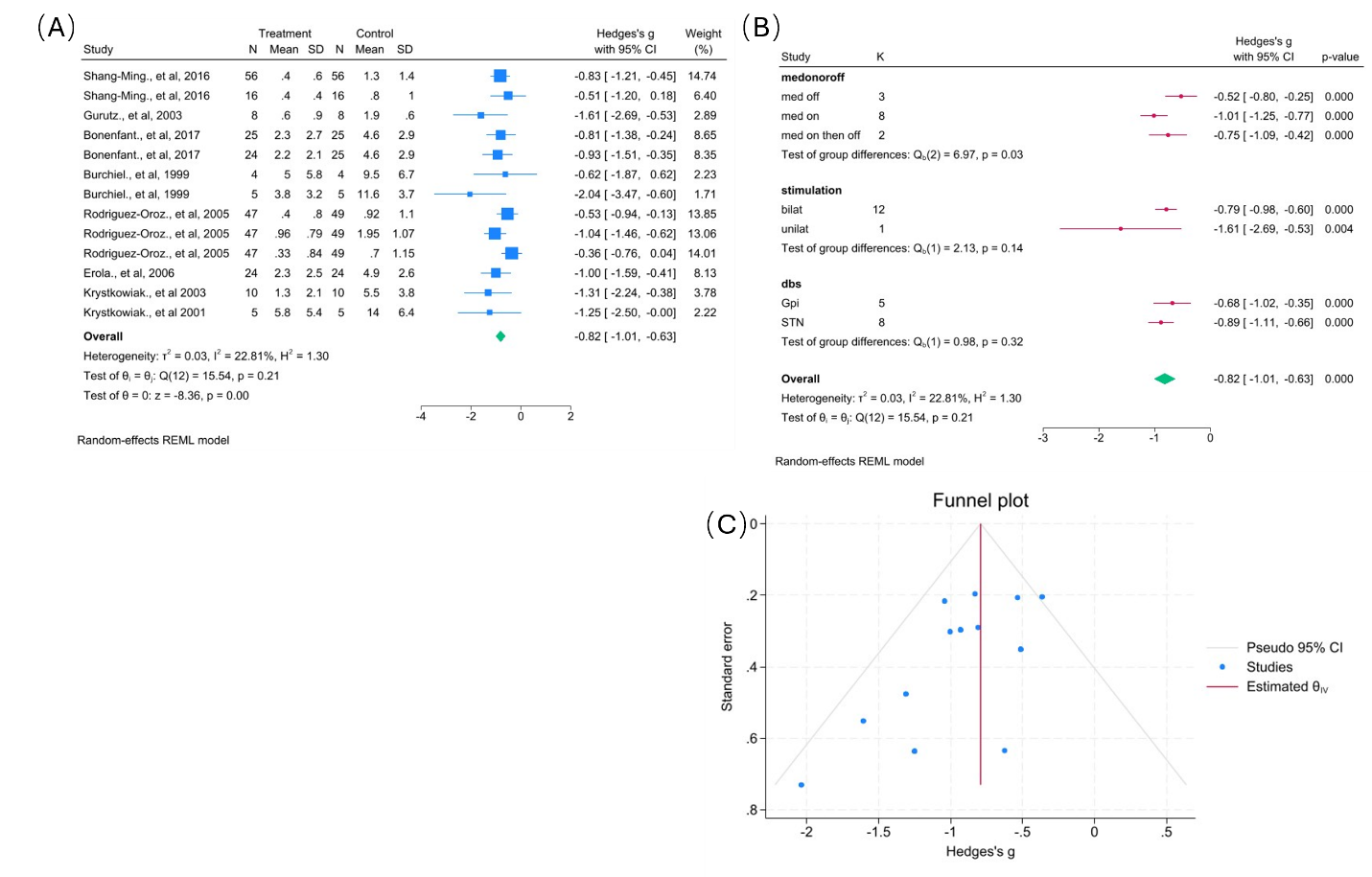


**Figure S5.** Meta-analysis of the effect of Deep Brain Stimulation on Dyskinesia after removing sources of heterogeneity. (A) Meta-analysis of the effect of Deep Brain Stimulation on Dyskinesia scores. (B) Subgroup analysis by medication status (med on vs. med off), stimulation type (bilateral vs. unilateral), and target site (GPi vs. STN). (C) Funnel plot assessing publication bias after removing sources of heterogeneity, with pseudo 95% CI. Diamonds represent the pooled effect estimates for overall and subgroup analyses. Data are represented as Hedges's g with 95% CI, using the random-effects REML model. Between-study heterogeneity is quantified by I^2^, with significance at p < 0.05. Test of homogeneity (θ_i_ = θ_j_) of study-specific effect sizes, with the chi-squared test statistic rejected if p < 0.05.

**6. Meta-analysis of Deep Brain Stimulation's Effects on Part 4**

One study (Thobois, et al, 2002) falls outside the confidence interval, suggesting it may contribute to the observed heterogeneity. After excluding this study, overall heterogeneity remained high (I² = 90.90%), indicating persistent discrepancies among researchers' findings (Figure S6A). The results of subgroup analyses are shown in Figure S6B. The funnel plot (Figure S6C) showed improved symmetry, suggesting a reduction in publication bias after excluding the outlier.


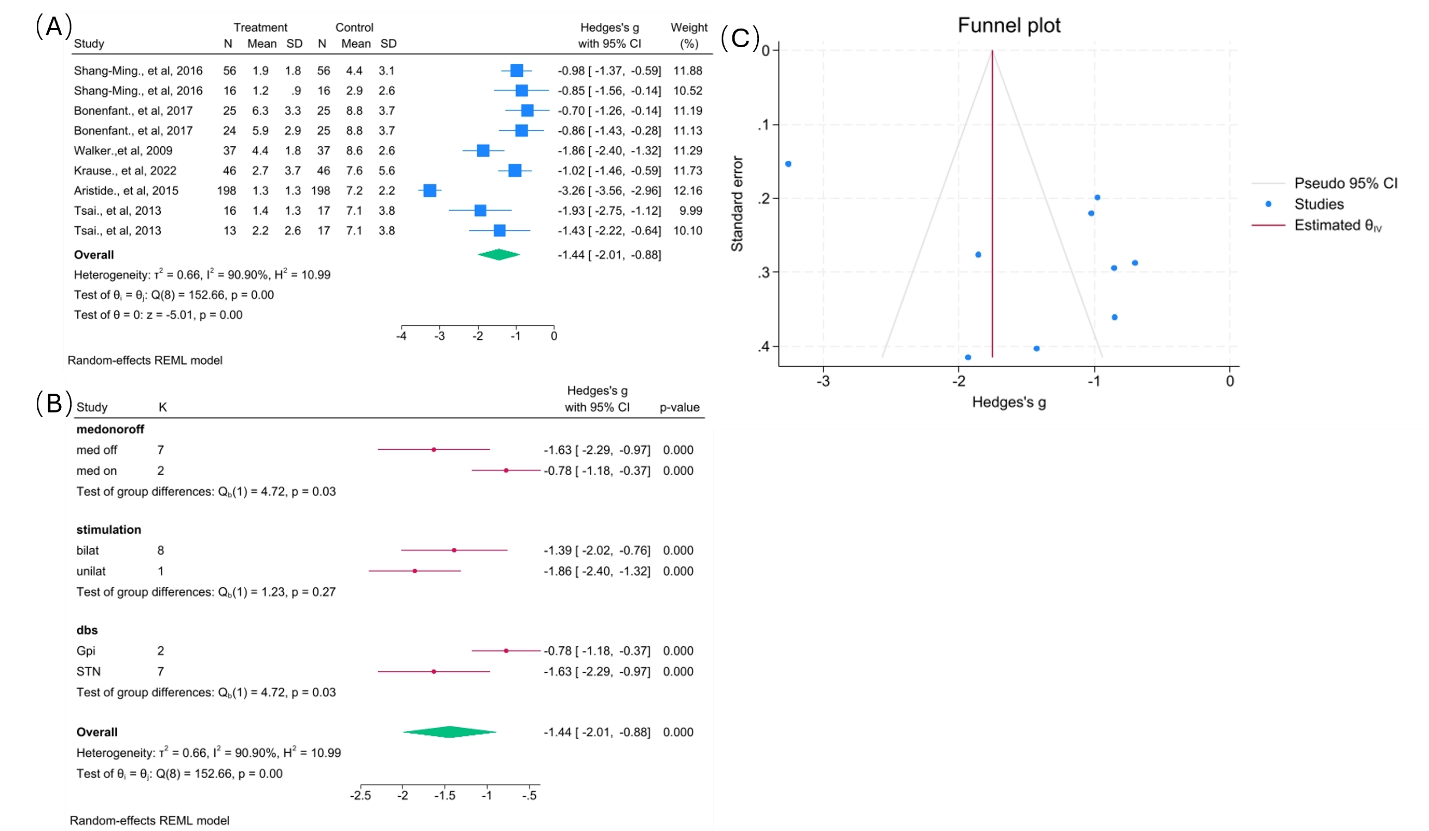


**Figure S6.** Meta-analysis of the effect of Deep Brain Stimulation on Part 4 after removing sources of heterogeneity. (A) Meta-analysis of the effect of Deep Brain Stimulation on Part 4 scores. (B) Subgroup analysis by medication status (med on vs. med off), stimulation type (bilateral vs. unilateral), and target site (GPi vs. STN). (C) Funnel plot assessing publication bias after removing sources of heterogeneity, with pseudo 95% CI. Diamonds represent the pooled effect estimates for overall and subgroup analyses. Data are represented as Hedges's g with 95% CI, using the random-effects REML model. Between-study heterogeneity is quantified by I^2^, with significance at p < 0.05. Test of homogeneity (θ_i_ = θ_j_) of study-specific effect sizes, with the chi-squared test statistic rejected if p < 0.05.

**7. Meta-analysis of Deep Brain Stimulation's Effects on Axial**

One study (Sobstyl, et al, 2014) falls outside the confidence interval, suggesting it may contribute to the observed heterogeneity. After removing this study, the overall heterogeneity did not decrease significantly (I^2^ = 84.55%), indicating that conflicting results were still reported by different researchers (Figure S7A). The results of subgroup analyses are shown in Figure S7B. The funnel plot (Figure S7C) showed improved symmetry, suggesting a reduction in publication bias after excluding the outlier.


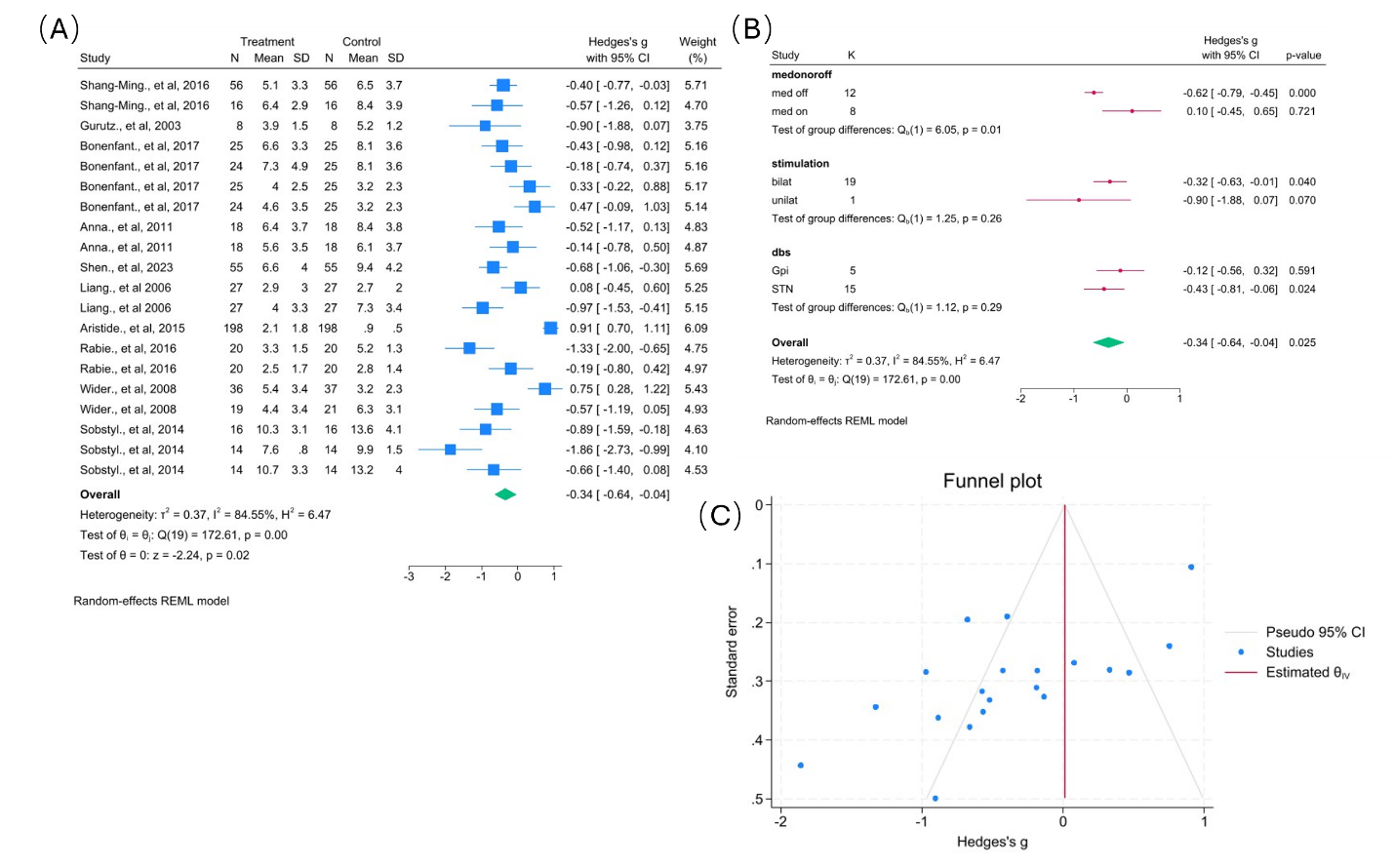


**Figure S7.** Meta-analysis of the effect of Deep Brain Stimulation on Axial after removing sources of heterogeneity. (A) Meta-analysis of the effect of Deep Brain Stimulation on Axial scores. (B) Subgroup analysis by medication status (med on vs. med off), stimulation type (bilateral vs. unilateral), and target site (GPi vs. STN). (C) Funnel plot assessing publication bias after removing sources of heterogeneity, with pseudo 95% CI. Diamonds represent the pooled effect estimates for overall and subgroup analyses. Data are represented as Hedges's g with 95% CI, using the random-effects REML model. Between-study heterogeneity is quantified by I^2^, with significance at p < 0.05. Test of homogeneity (θ_i_ = θ_j_) of study-specific effect sizes, with the chi-squared test statistic rejected if p < 0.05.

**8. Meta-analysis of Deep Brain Stimulation's Effects on Speech**

One study (Doshi, et al, 2003) falls outside the confidence interval, suggesting it may contribute to the observed heterogeneity. After removing this study, the overall heterogeneity slightly decreased (I^2^ = 78.13%, p < 0.05), but the effect remained non-significant (WMD = -0.10, 95% CI: -0.33 to 0.13; p > 0.05; Figure S8A). The results of subgroup analyses are shown in Figure S8B. The funnel plot (Figure S8C) showed improved symmetry, suggesting a reduction in publication bias after excluding the outlier.


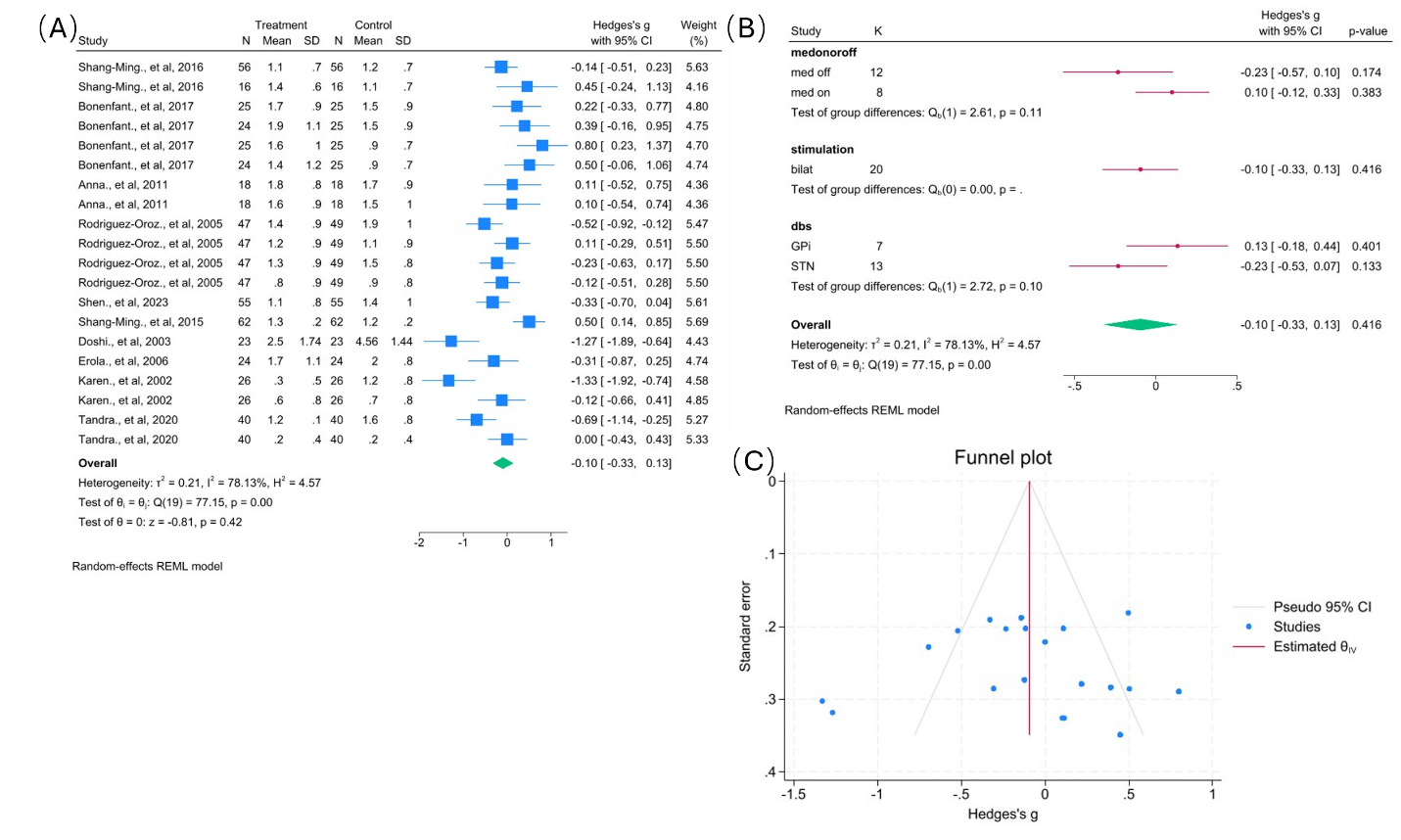


**Figure S8.** Meta-analysis of the effect of Deep Brain Stimulation on Speech after removing sources of heterogeneity. (A) Meta-analysis of the effect of Deep Brain Stimulation on Speech scores. (B) Subgroup analysis by medication status (med on vs. med off), stimulation type (bilateral vs. unilateral), and target site (GPi vs. STN). (C) Funnel plot assessing publication bias after removing sources of heterogeneity, with pseudo 95% CI. Diamonds represent the pooled effect estimates for overall and subgroup analyses. Data are represented as Hedges's g with 95% CI, using the random-effects REML model. Between-study heterogeneity is quantified by I^2^, with significance at p < 0.05. Test of homogeneity (θ_i_ = θ_j_) of study-specific effect sizes, with the chi-squared test statistic rejected if p < 0.05.

**9. Meta-analysis of Deep Brain Stimulation's Effects on Part 1**

After removing the identified outliers, the overall effect size slightly increased, showing a significant improvement (WMD = -0.24, 95% CI: -0.40 to -0.08; p < 0.05; Figure S15A). The heterogeneity substantially decreased (I^2^ = 0.00%, p > 0.05), indicating that the remaining studies were highly consistent (Figure S9A). Subgroup analysis also revealed highly consistent (Figure S9B). The funnel plot (Figure S9C) showed improved symmetry, suggesting reduced publication bias. These findings suggest that the observed heterogeneity in the original analysis was primarily driven by a few studies, and after their exclusion, the effect of Deep Brain Stimulation on Part 1 scores became statistically significant.


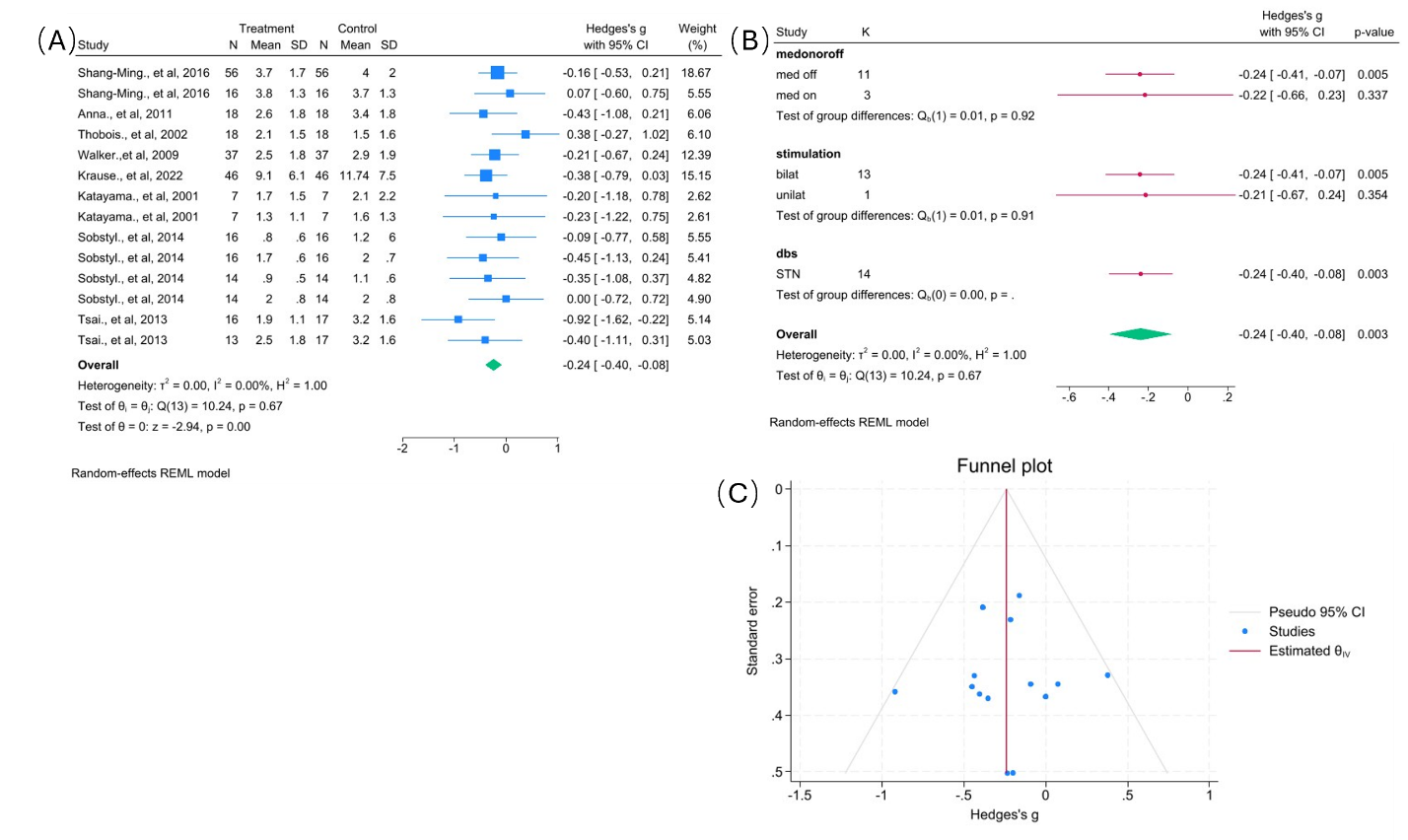


**Figure S9.** Meta-analysis of the effect of Deep Brain Stimulation on Part 1 after removing sources of heterogeneity. (A) Meta-analysis of the effect of Deep Brain Stimulation on Part 1 scores. (B) Subgroup analysis by medication status (med on vs. med off), stimulation type (bilateral vs. unilateral), and target site (GPi vs. STN). (C) Funnel plot assessing publication bias after removing sources of heterogeneity, with pseudo 95% CI. Diamonds represent the pooled effect estimates for overall and subgroup analyses. Data are represented as Hedges's g with 95% CI, using the random-effects REML model. Between-study heterogeneity is quantified by I^2^, with significance at p < 0.05. Test of homogeneity (θ_i_ = θ_j_) of study-specific effect sizes, with the chi-squared test statistic rejected if p < 0.05.

**10. Meta-analysis of Deep Brain Stimulation's Effects on Part 2**

However, four studies fall outside the confidence interval, suggesting they may contribute to the observed heterogeneity. After removing these four studies, the overall heterogeneity did not decrease significantly (I^2^ = 86.53%), indicating that conflicting results were still reported by different researchers (Figure S10A). The results of subgroup analyses are shown in Figure S10B. The funnel plot (Figure S10C) showed improved symmetry, suggesting a reduction in publication bias after excluding the outliers.
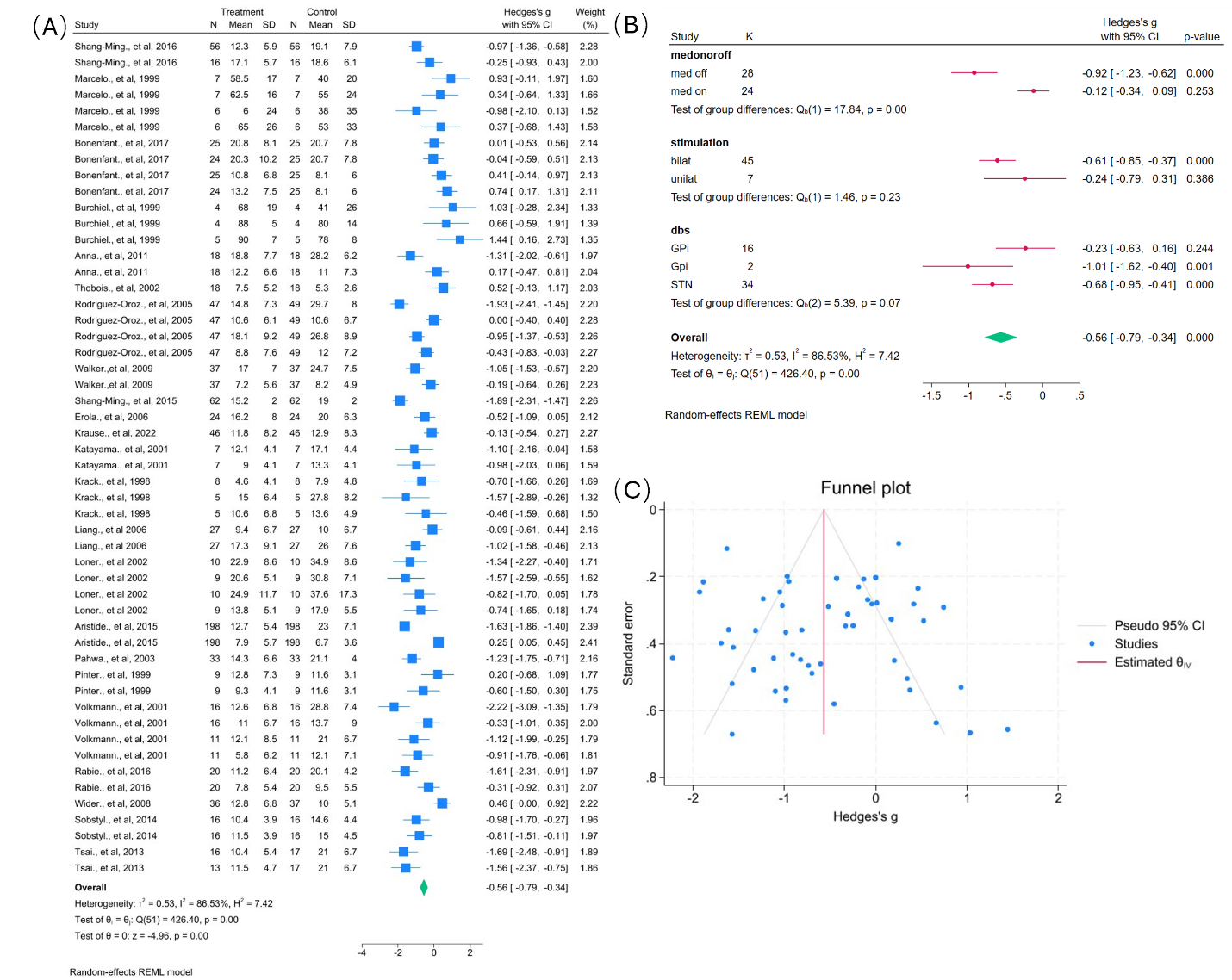


**Figure S10**. Meta-analysis of the effect of Deep Brain Stimulation on Part 2 after removing sources of heterogeneity. (A) Meta-analysis of the effect of Deep Brain Stimulation on Part 2 scores. (B) Subgroup analysis by medication status (med on vs. med off), stimulation type (bilateral vs. unilateral), and target site (GPi vs. STN). (C) Funnel plot assessing publication bias after removing sources of heterogeneity, with pseudo 95% CI. Diamonds represent the pooled effect estimates for overall and subgroup analyses. Data are represented as Hedges's g with 95% CI, using the random-effects REML model. Between-study heterogeneity is quantified by I^2^, with significance at p < 0.05. Test of homogeneity (θ_i_ = θ_j_) of study-specific effect sizes, with the chi-squared test statistic rejected if p < 0.05.

**11. Meta-analysis of Deep Brain Stimulation's Effects on UPDRS-TOTAL score**

two studies fall outside the confidence interval, suggesting they may contribute to the observed heterogeneity. After removing these two studies, the overall heterogeneity did not decrease significantly (I^2^ = 83.71%), indicating that conflicting results were still reported by different researchers (Figure S11A). The results of subgroup analyses are shown in Figure S11B. The funnel plot (Figure S11C) showed improved symmetry, suggesting a reduction in publication bias after excluding the outliers.


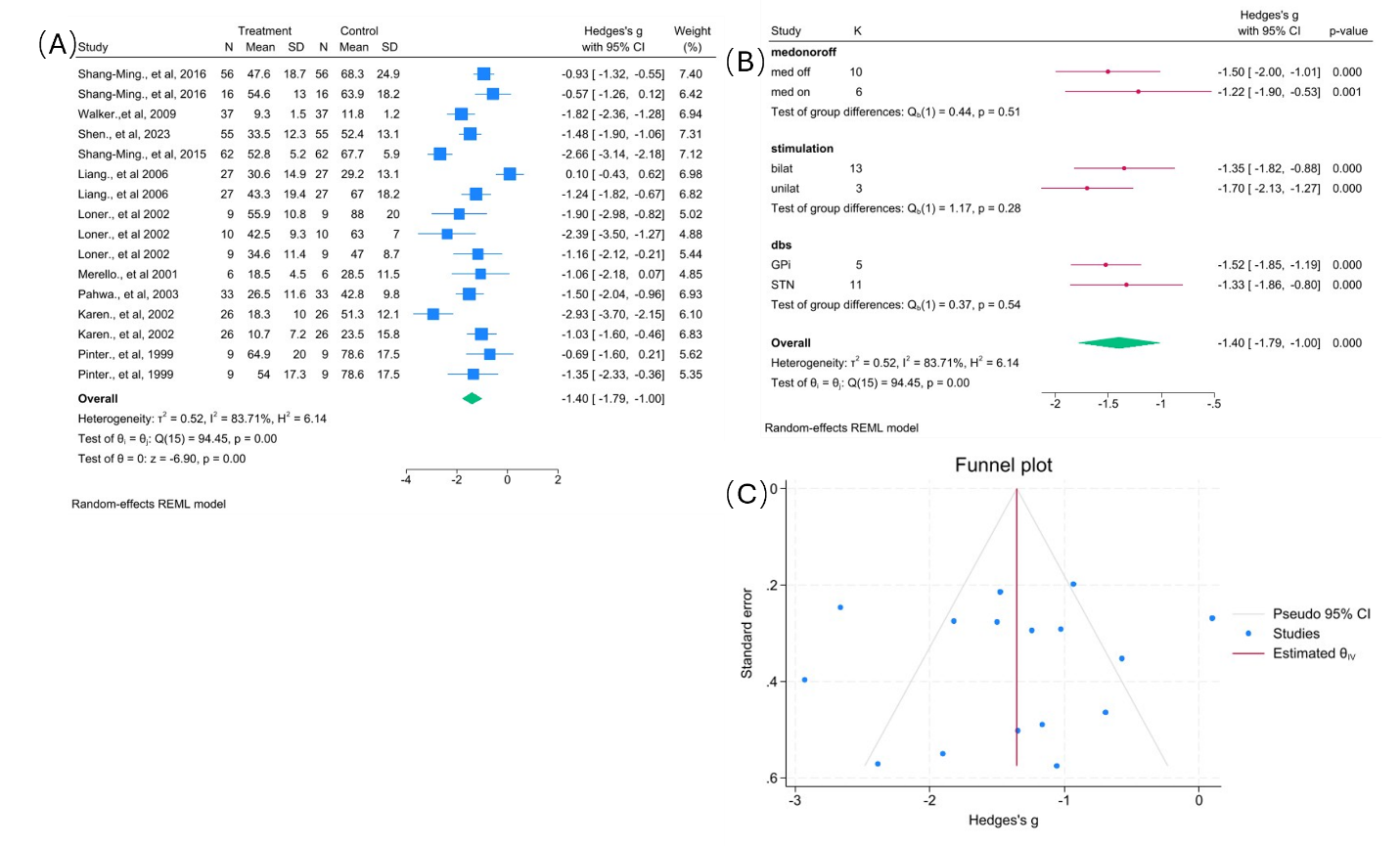


**Figure S11.** Meta-analysis of the effect of Deep Brain Stimulation on UPDRS-TOTAL after removing sources of heterogeneity. (A) Meta-analysis of the effect of Deep Brain Stimulation on UPDRS-TOTAL scores. (B) Subgroup analysis by medication status (med on vs. med off), stimulation type (bilateral vs. unilateral), and target site (GPi vs. STN). (C) Funnel plot assessing publication bias after removing sources of heterogeneity, with pseudo 95% CI. Diamonds represent the pooled effect estimates for overall and subgroup analyses. Data are represented as Hedges's g with 95% CI, using the random-effects REML model. Between-study heterogeneity is quantified by I^2^, with significance at p < 0.05. Test of homogeneity (θ_i_ = θ_j_) of study-specific effect sizes, with the chi-squared test statistic rejected if p < 0.05.

**Supplemental Table 1.** Search strategies in used each database.

| **Database** | **Search terms** |
| --- | --- |
| PubMed | ((((((((((((((((((((((((((((((((((((((((Dyskinesia) OR (Dyskinesia)) OR (Abnormal Movement)) OR (Movement, Abnormal)) OR (Movements, Abnormal)) OR (Asterixis)) OR (Ballismus)) OR (Hemiballismus)) OR (Hemiballism)) OR (Involuntary Movements)) OR (Involuntary Movement)) OR (Movement, Involuntary)) OR (Movement, Involuntary)) OR (Movements, Involuntary)) OR (Orofacial Dyskinesia)) OR (Dyskinesia, Orofacial)) OR (Dyskinesias, Orofacial)) OR (Orofacial Dyskinesias)) OR (Lingual-Facial-Buccal Dyskinesia)) OR (Dyskinesia, Lingual-Facial-Buccal)) OR (Dyskinesias, Lingual-Facial-Buccal)) OR (Lingual Facial Buccal Dyskinesia)) OR (Lingual-Facial-Buccal Dyskinesias)) OR (Tardive Oral Dyskinesia)) OR (Tardive Oral Dyskinesias)) OR (Linguofacial Dyskinesia)) OR (Dyskinesia, Linguofacial)) OR (Dyskinesias, Linguofacial)) OR (Linguofacial Dyskinesias)) OR (Oral-Facial Dyskinesia)) OR (Dyskinesia, Oral-Facial)) OR (Dyskinesias, Oral-Facial)) OR (Oral Facial Dyskinesia)) OR (Oral-Facial Dyskinesias)) OR (Oral Dyskinesia)) OR (Dyskinesia, Oral)) OR (Dyskinesias, Oral)) OR (Oral Dyskinesias)) OR ("Dyskinesias"[Mesh])) AND ((((((((((((Idiopathic Parkinson's Disease) OR (Lewy Body Parkinson's Disease)) OR (Parkinson's Disease, Idiopathic)) OR (Parkinson's Disease, Lewy Body)) OR (Paralysis Agitans)) OR (Parkinson's Disease)) OR (Idiopathic Parkinson Disease)) OR (Lewy Body Parkinson Disease)) OR (Primary Parkinsonism)) OR (Parkinsonism, Primary)) OR (Parkinson Disease, Idiopathic)) OR ("Parkinson Disease"[Mesh]))) AND (((((((Brain Stimulations, Deep) OR (Deep Brain Stimulations)) OR (Stimulation, Deep Brain)) OR (Stimulations, Deep Brain)) OR (Brain Stimulation, Deep)) OR (Electrical Stimulation of the Brain)) OR ("Deep Brain Stimulation"[Mesh])). The search method was applied to all databases. |
| Embase | ((((((((((((((((((((((((((((((((((((((((Dyskinesia) OR (Dyskinesia)) OR (Abnormal Movement)) OR (Movement, Abnormal)) OR (Movements, Abnormal)) OR (Asterixis)) OR (Ballismus)) OR (Hemiballismus)) OR (Hemiballism)) OR (Involuntary Movements)) OR (Involuntary Movement)) OR (Movement, Involuntary)) OR (Movement, Involuntary)) OR (Movements, Involuntary)) OR (Orofacial Dyskinesia)) OR (Dyskinesia, Orofacial)) OR (Dyskinesias, Orofacial)) OR (Orofacial Dyskinesias)) OR (Lingual-Facial-Buccal Dyskinesia)) OR (Dyskinesia, Lingual-Facial-Buccal)) OR (Dyskinesias, Lingual-Facial-Buccal)) OR (Lingual Facial Buccal Dyskinesia)) OR (Lingual-Facial-Buccal Dyskinesias)) OR (Tardive Oral Dyskinesia)) OR (Tardive Oral Dyskinesias)) OR (Linguofacial Dyskinesia)) OR (Dyskinesia, Linguofacial)) OR (Dyskinesias, Linguofacial)) OR (Linguofacial Dyskinesias)) OR (Oral-Facial Dyskinesia)) OR (Dyskinesia, Oral-Facial)) OR (Dyskinesias, Oral-Facial)) OR (Oral Facial Dyskinesia)) OR (Oral-Facial Dyskinesias)) OR (Oral Dyskinesia)) OR (Dyskinesia, Oral)) OR (Dyskinesias, Oral)) OR (Oral Dyskinesias)) OR ("Dyskinesias"[Mesh])) AND ((((((((((((Idiopathic Parkinson's Disease) OR (Lewy Body Parkinson's Disease)) OR (Parkinson's Disease, Idiopathic)) OR (Parkinson's Disease, Lewy Body)) OR (Paralysis Agitans)) OR (Parkinson's Disease)) OR (Idiopathic Parkinson Disease)) OR (Lewy Body Parkinson Disease)) OR (Primary Parkinsonism)) OR (Parkinsonism, Primary)) OR (Parkinson Disease, Idiopathic)) OR ("Parkinson Disease"[Mesh]))) AND (((((((Brain Stimulations, Deep) OR (Deep Brain Stimulations)) OR (Stimulation, Deep Brain)) OR (Stimulations, Deep Brain)) OR (Brain Stimulation, Deep)) OR (Electrical Stimulation of the Brain)) OR ("Deep Brain Stimulation"[Mesh])). The search method was applied to all databases. |
| the Cochrane Central Register of Controlled Trials (CENTRAL) | ((((((((((((((((((((((((((((((((((((((((Dyskinesia) OR (Dyskinesia)) OR (Abnormal Movement)) OR (Movement, Abnormal)) OR (Movements, Abnormal)) OR (Asterixis)) OR (Ballismus)) OR (Hemiballismus)) OR (Hemiballism)) OR (Involuntary Movements)) OR (Involuntary Movement)) OR (Movement, Involuntary)) OR (Movement, Involuntary)) OR (Movements, Involuntary)) OR (Orofacial Dyskinesia)) OR (Dyskinesia, Orofacial)) OR (Dyskinesias, Orofacial)) OR (Orofacial Dyskinesias)) OR (Lingual-Facial-Buccal Dyskinesia)) OR (Dyskinesia, Lingual-Facial-Buccal)) OR (Dyskinesias, Lingual-Facial-Buccal)) OR (Lingual Facial Buccal Dyskinesia)) OR (Lingual-Facial-Buccal Dyskinesias)) OR (Tardive Oral Dyskinesia)) OR (Tardive Oral Dyskinesias)) OR (Linguofacial Dyskinesia)) OR (Dyskinesia, Linguofacial)) OR (Dyskinesias, Linguofacial)) OR (Linguofacial Dyskinesias)) OR (Oral-Facial Dyskinesia)) OR (Dyskinesia, Oral-Facial)) OR (Dyskinesias, Oral-Facial)) OR (Oral Facial Dyskinesia)) OR (Oral-Facial Dyskinesias)) OR (Oral Dyskinesia)) OR (Dyskinesia, Oral)) OR (Dyskinesias, Oral)) OR (Oral Dyskinesias)) OR ("Dyskinesias"[Mesh])) AND ((((((((((((Idiopathic Parkinson's Disease) OR (Lewy Body Parkinson's Disease)) OR (Parkinson's Disease, Idiopathic)) OR (Parkinson's Disease, Lewy Body)) OR (Paralysis Agitans)) OR (Parkinson's Disease)) OR (Idiopathic Parkinson Disease)) OR (Lewy Body Parkinson Disease)) OR (Primary Parkinsonism)) OR (Parkinsonism, Primary)) OR (Parkinson Disease, Idiopathic)) OR ("Parkinson Disease"[Mesh]))) AND (((((((Brain Stimulations, Deep) OR (Deep Brain Stimulations)) OR (Stimulation, Deep Brain)) OR (Stimulations, Deep Brain)) OR (Brain Stimulation, Deep)) OR (Electrical Stimulation of the Brain)) OR ("Deep Brain Stimulation"[Mesh])). The search method was applied to all databases. |
| Ovid MEDLINE | ((((((((((((((((((((((((((((((((((((((((Dyskinesia) OR (Dyskinesia)) OR (Abnormal Movement)) OR (Movement, Abnormal)) OR (Movements, Abnormal)) OR (Asterixis)) OR (Ballismus)) OR (Hemiballismus)) OR (Hemiballism)) OR (Involuntary Movements)) OR (Involuntary Movement)) OR (Movement, Involuntary)) OR (Movement, Involuntary)) OR (Movements, Involuntary)) OR (Orofacial Dyskinesia)) OR (Dyskinesia, Orofacial)) OR (Dyskinesias, Orofacial)) OR (Orofacial Dyskinesias)) OR (Lingual-Facial-Buccal Dyskinesia)) OR (Dyskinesia, Lingual-Facial-Buccal)) OR (Dyskinesias, Lingual-Facial-Buccal)) OR (Lingual Facial Buccal Dyskinesia)) OR (Lingual-Facial-Buccal Dyskinesias)) OR (Tardive Oral Dyskinesia)) OR (Tardive Oral Dyskinesias)) OR (Linguofacial Dyskinesia)) OR (Dyskinesia, Linguofacial)) OR (Dyskinesias, Linguofacial)) OR (Linguofacial Dyskinesias)) OR (Oral-Facial Dyskinesia)) OR (Dyskinesia, Oral-Facial)) OR (Dyskinesias, Oral-Facial)) OR (Oral Facial Dyskinesia)) OR (Oral-Facial Dyskinesias)) OR (Oral Dyskinesia)) OR (Dyskinesia, Oral)) OR (Dyskinesias, Oral)) OR (Oral Dyskinesias)) OR ("Dyskinesias"[Mesh])) AND ((((((((((((Idiopathic Parkinson's Disease) OR (Lewy Body Parkinson's Disease)) OR (Parkinson's Disease, Idiopathic)) OR (Parkinson's Disease, Lewy Body)) OR (Paralysis Agitans)) OR (Parkinson's Disease)) OR (Idiopathic Parkinson Disease)) OR (Lewy Body Parkinson Disease)) OR (Primary Parkinsonism)) OR (Parkinsonism, Primary)) OR (Parkinson Disease, Idiopathic)) OR ("Parkinson Disease"[Mesh]))) AND (((((((Brain Stimulations, Deep) OR (Deep Brain Stimulations)) OR (Stimulation, Deep Brain)) OR (Stimulations, Deep Brain)) OR (Brain Stimulation, Deep)) OR (Electrical Stimulation of the Brain)) OR ("Deep Brain Stimulation"[Mesh])). The search method was applied to all databases. |
| Web of Science | ((((((((((((((((((((((((((((((((((((((((Dyskinesia) OR (Dyskinesia)) OR (Abnormal Movement)) OR (Movement, Abnormal)) OR (Movements, Abnormal)) OR (Asterixis)) OR (Ballismus)) OR (Hemiballismus)) OR (Hemiballism)) OR (Involuntary Movements)) OR (Involuntary Movement)) OR (Movement, Involuntary)) OR (Movement, Involuntary)) OR (Movements, Involuntary)) OR (Orofacial Dyskinesia)) OR (Dyskinesia, Orofacial)) OR (Dyskinesias, Orofacial)) OR (Orofacial Dyskinesias)) OR (Lingual-Facial-Buccal Dyskinesia)) OR (Dyskinesia, Lingual-Facial-Buccal)) OR (Dyskinesias, Lingual-Facial-Buccal)) OR (Lingual Facial Buccal Dyskinesia)) OR (Lingual-Facial-Buccal Dyskinesias)) OR (Tardive Oral Dyskinesia)) OR (Tardive Oral Dyskinesias)) OR (Linguofacial Dyskinesia)) OR (Dyskinesia, Linguofacial)) OR (Dyskinesias, Linguofacial)) OR (Linguofacial Dyskinesias)) OR (Oral-Facial Dyskinesia)) OR (Dyskinesia, Oral-Facial)) OR (Dyskinesias, Oral-Facial)) OR (Oral Facial Dyskinesia)) OR (Oral-Facial Dyskinesias)) OR (Oral Dyskinesia)) OR (Dyskinesia, Oral)) OR (Dyskinesias, Oral)) OR (Oral Dyskinesias)) OR ("Dyskinesias"[Mesh])) AND ((((((((((((Idiopathic Parkinson's Disease) OR (Lewy Body Parkinson's Disease)) OR (Parkinson's Disease, Idiopathic)) OR (Parkinson's Disease, Lewy Body)) OR (Paralysis Agitans)) OR (Parkinson's Disease)) OR (Idiopathic Parkinson Disease)) OR (Lewy Body Parkinson Disease)) OR (Primary Parkinsonism)) OR (Parkinsonism, Primary)) OR (Parkinson Disease, Idiopathic)) OR ("Parkinson Disease"[Mesh]))) AND (((((((Brain Stimulations, Deep) OR (Deep Brain Stimulations)) OR (Stimulation, Deep Brain)) OR (Stimulations, Deep Brain)) OR (Brain Stimulation, Deep)) OR (Electrical Stimulation of the Brain)) OR ("Deep Brain Stimulation"[Mesh])). The search method was applied to all databases. |
